# Genome Wide Expression Profiling of Cancer Cell Lines Cultured in Microgravity Reveals Significant Dysregulation of Cell Cycle and MicroRNA Gene Networks

**DOI:** 10.1371/journal.pone.0135958

**Published:** 2015-08-21

**Authors:** Prasanna Vidyasekar, Pavithra Shyamsunder, Rajpranap Arun, Rajalakshmi Santhakumar, Nand Kishore Kapadia, Ravi Kumar, Rama Shanker Verma

**Affiliations:** 1 Stem cell and Molecular Biology laboratory, Department of Biotechnology, Indian Institute of Technology Madras, Chennai, India; 2 Department of cardiothoracic Surgery, Global Hospital, Perumbakkam, Chennai, India; 3 Department of cardiology, Fortis Malar Hospital, Adyar, Chennai, India; Peking Union Medical College Hospital, CHINA

## Abstract

Zero gravity causes several changes in metabolic and functional aspects of the human body and experiments in space flight have demonstrated alterations in cancer growth and progression. This study reports the genome wide expression profiling of a colorectal cancer cell line-DLD-1, and a lymphoblast leukemic cell line-MOLT-4, under simulated microgravity in an effort to understand central processes and cellular functions that are dysregulated among both cell lines. Altered cell morphology, reduced cell viability and an aberrant cell cycle profile in comparison to their static controls were observed in both cell lines under microgravity. The process of cell cycle in DLD-1 cells was markedly affected with reduced viability, reduced colony forming ability, an apoptotic population and dysregulation of cell cycle genes, oncogenes, and cancer progression and prognostic markers. DNA microarray analysis revealed 1801 (upregulated) and 2542 (downregulated) genes (>2 fold) in DLD-1 cultures under microgravity while MOLT-4 cultures differentially expressed 349 (upregulated) and 444 (downregulated) genes (>2 fold) under microgravity. The loss in cell proliferative capacity was corroborated with the downregulation of the cell cycle process as demonstrated by functional clustering of DNA microarray data using gene ontology terms. The genome wide expression profile also showed significant dysregulation of post transcriptional gene silencing machinery and multiple microRNA host genes that are potential tumor suppressors and proto-oncogenes including *MIR22HG*, *MIR17HG* and *MIR21HG*. The *MIR22HG*, a tumor-suppressor gene was one of the highest upregulated genes in the microarray data showing a 4.4 log fold upregulation under microgravity. Real time PCR validated the dysregulation in the host gene by demonstrating a 4.18 log fold upregulation of the miR-22 microRNA. Microarray data also showed dysregulation of direct targets of miR-22, *SP1*, *CDK6* and *CCNA2*.

## Introduction

Microgravity on space flights has been shown to affect the physiology of a cell considerably [1]. Normal gravity (1 g) affects 2-Dimensional culture by depositing cells on the surface of the tissue culture plate (TCP) where anchorage-dependent cells adhere and proliferate as a monolayer with very limited cell–cell interactions. The weightlessness and reduced acceleration (less than 1 g) in space, removes the effect of gravity, allowing cell cultures in space to have unhindered movement of the culture medium, a shear free environment and, as cells are not bound by any directional force, unrestricted movement of cells within the medium. Under such conditions cells tend to coalesce and form aggregates creating three dimensional (3D) environments where they interact on multiple planes [2]. The effect of reduced gravity is not restricted to changes in culture conditions as the unique environment can produce changes in the fundamental physiology of the cell. While the mechanism of action of how gravity, or the lack of it, affects molecular and cellular functions is still unclear, it has been established that microgravity or zero gravity affects vital processes of the cell and importantly, microgravity has been shown to alter cancer growth and progression [3–5]. However, different cancers respond differently to microgravity by losing or enhancing cellular processes and functions. In this study we cultured cell lines representative of solid and hematological tumors—DLD-1, MOLT-4 and HL-60 in a rotating cell culture system (RCCS) that simulated microgravity. The RCCS is a mechanical system that simulates reduced gravity on earth by canceling the directional vector through constant rotation of a High Aspect Ratio Vessel (HARV). This maintains cells in a constant free fall and a shear free environment allowing cells to coalesce and form 3D aggregates [2]. These aggregates are maintained in free fall and experience conditions of reduced gravity for the remainder of the culture period. We hypothesized that physiological changes to the cell functions such as cell proliferation and viability could be corroborated with changes in fundamental processes of the cell such as gene expression. To relate physiological changes such as an altered cell cycle profile with dysregulation of gene expression, real time PCR analysis for cell cycle genes, oncogenes and cancer development and progression markers was carried out. Genome wide expression profiling by DNA microarray of these cell lines cultured under microgravity revealed the dysregulation of several pathways in cancer and importantly, corroborated with observed physiological changes to the cell. We also used the gene expression profile to investigate dysregulation in pathways central to cancer such as the Notch signaling system and dysregulation in post transcriptional gene silencing machinery. The gene expression profile also revealed dysregulation of microRNA host genes in microgravity including the significant tumor suppressor, miR-22 in DLD-1.

## Materials and Methods

### Cell culture

DLD-1 is an epithelial, adherent cell line derived from a colorectal adenocarcinoma (Dukes type C). MOLT-4 is a T lymphoblast, suspension cell line derived from an acute lymphoblastic leukemia while the HL-60 cell line is a promyeloblast derived from acute promyelocytic leukemia. Cell lines were procured from the National centre for cell science, Pune, India and were maintained in DMEM-F12 (DLD-1) or RPMI1640 (MOLT-4, HL-60) medium supplemented with 10% fetal bovine serum (Life Technologies, USA) at 37°C in a humidified 5% CO2 incubator in 25mm^3^ tissue culture plates (TCP) and in 10ml^3^ high aspect ratio vessels (HARV) within a rotating cell culture system (RCCS). The cell lines were introduced into the 10ml HARV through 5ml syringes and a rotating speed of 27 revolutions per minute (RPM) was standardized based on the aggregation of DLD-1 cells loaded at 0.5 x 10^6^ cells within 24 to 48 hours. Cells were grown in HARV for a maximum of 72 hours with additional medium injected into the HARV every 16 to 24 hrs to prevent foaming or air bubbles. Contents of the HARV were transferred to 60mm TCP, without dissociating cell aggregates, for routine micrographic observation and other cell based assays by using 10ml pipettes. 0.25% Trypsin-EDTA was used for dissociation of cell aggregates and monolayer cultures when required.

### Total RNA extraction and cDNA conversion

Total RNA was extracted from cells using RNeasy kit (Qiagen, Germany). 2 × 10^6^ cells were centrifuged and washed twice with PBS. RNA was isolated from these cells as per the manufacturer's instructions. 1.5 μg of total RNA was converted to cDNA using MMLV-RT (Thermo scientific, USA) and Oligo-dT primers (NEB, USA). miRNA conversion to cDNA was carried out using stem-loop reverse transcriptase (RT) primers without dithiothreitol (DTT) and the RNA denaturation step to maintain integrity of the stem-loop primer.

### Microarray analysis

Microarray analysis was performed with RNA samples from DLD-1 and MOLT-4 cell lines grown under microgravity and under static conditions in replicates. Expression data for each sample was obtained on the Affymetrix GeneChip Human Primeview Array. Hybridization was carried out for a duration of 16 hours at 60 rpm at 48°C and scanned on the GeneChip microarray Scanner 3000 7G. Raw data was extracted after scanning of slides and raw data sets were analyzed using GeneSpring GX 12.6 software followed by differential gene expression (DE), fold change & cluster analysis.

### Gene ontology analysis

The DE genes were studied for their overabundance in different Gene ontology (GO) terms as well as pathways using the microarray analysis software DAVID (The Database for Annotation, Visualization and Integrated Discovery) [6]. Two tools in the DAVID program were used, gene functional classification tool and the functional annotation clustering tool. The DAVID functional annotation tool was used to highlight relevant GO terms associated with the submitted gene list by grouping similar, redundant, and heterogeneous annotation contents from the same or different resources into annotation groups based on the hypothesis that similar annotations should have similar gene members. Gene enrichment is based on set of submitted genes that are highly associated with certain terms, which is statistically measured by Fisher Exact in DAVID system. In this study, we used the group enrichment score which is the geometric mean of all p-values of individual members in a corresponding annotation cluster. A higher score indicates a highly enriched cluster which in turn indicates the biological significance of the cluster in the list.

### Real time PCR amplification

Real time PCR was carried out using SYBR green Real time PCR kit from Qiagen on an Eppendorf mastercycler, ep realplex (Eppendorf, Germany). Relative mRNA expression was determined by normalization to the expression of a housekeeping gene, beta-actin and U6 for microRNA gene expression. Primer list is provided in S1 Table.

### Western blotting

Cells were lysed with Radio Immuno Precipitation Assay (RIPA) buffer, mixed with Laemmli sample buffer (1×) and boiled. Proteins were subjected to 12% SDS-PAGE and electroblotted onto BioRad, 0.22 μM nitrocellulose membrane (BioRad Laboratories, USA). Membrane was blocked with Tris-buffered saline plus 0.2% Tween 20 (TBS-T) containing 3% BSA (Sigma Aldrich, USA) followed by primary antibody incubation overnight and washing with TBS-T buffer. Secondary antibody (anti-mouse, HRP conjugate, 1:10000 Sigma Aldrich USA) diluted in blocking buffer was incubated for 1 h at room temperature and washed again with TBS-T. Antibody-reactive proteins were detected by means of enhanced chemiluminescence, Amersham ECL Plus western blotting detection reagents (GE health care, UK). Antibody details are provided in S1 Table.

### Flow cytometry for cell cycle analysis

The cells were harvested and washed in PBS before fixation in cold 70% ethanol which was added drop wise to the pellet while vortexing. Cells were fixed for 30 min at 4°C. Fixed cells were washed twice in PBS and spun at 250g in a centrifuge. Cells were incubated with 50 μl of a 100 μg/ml stock of RNase and 200 μl Propidium Iodide (from 50 μg/ml stock solution). A BD FACSCalibur (USA) flow cytometer was used to analyze the cell population for cell cycle changes.

### CFU- assay

Cells were cultured in methyl cellulose at 10X of final concentration (0.3 mL of cells to 3 mL of Methyl cellulose). Tubes were vortexed to ensure cells and components were thoroughly mixed. Methyl cellulose was dispensed using a 3cc syringe and evenly spread in the dish by gentle swirling. Cultures were incubated at 37°C, 5% CO2 in air and ≥95% humidity. Colonies were then stained with crystal violet (0.5mg/ml in 1% methanol) for 20 minutes and air dried after washing in distilled water. Stained colonies were visualized under a Nikon eclipse Ti phase contrast microscope.

### Statistical analysis

All experiments were carried out in replicates and results were expressed as mean ± S.D. Statistical significance was calculated by using student t-test with the Prism 5 program (GraphPad software, USA).

## Results and Discussion

### Culture of cells in microgravity

**Fig 1:**
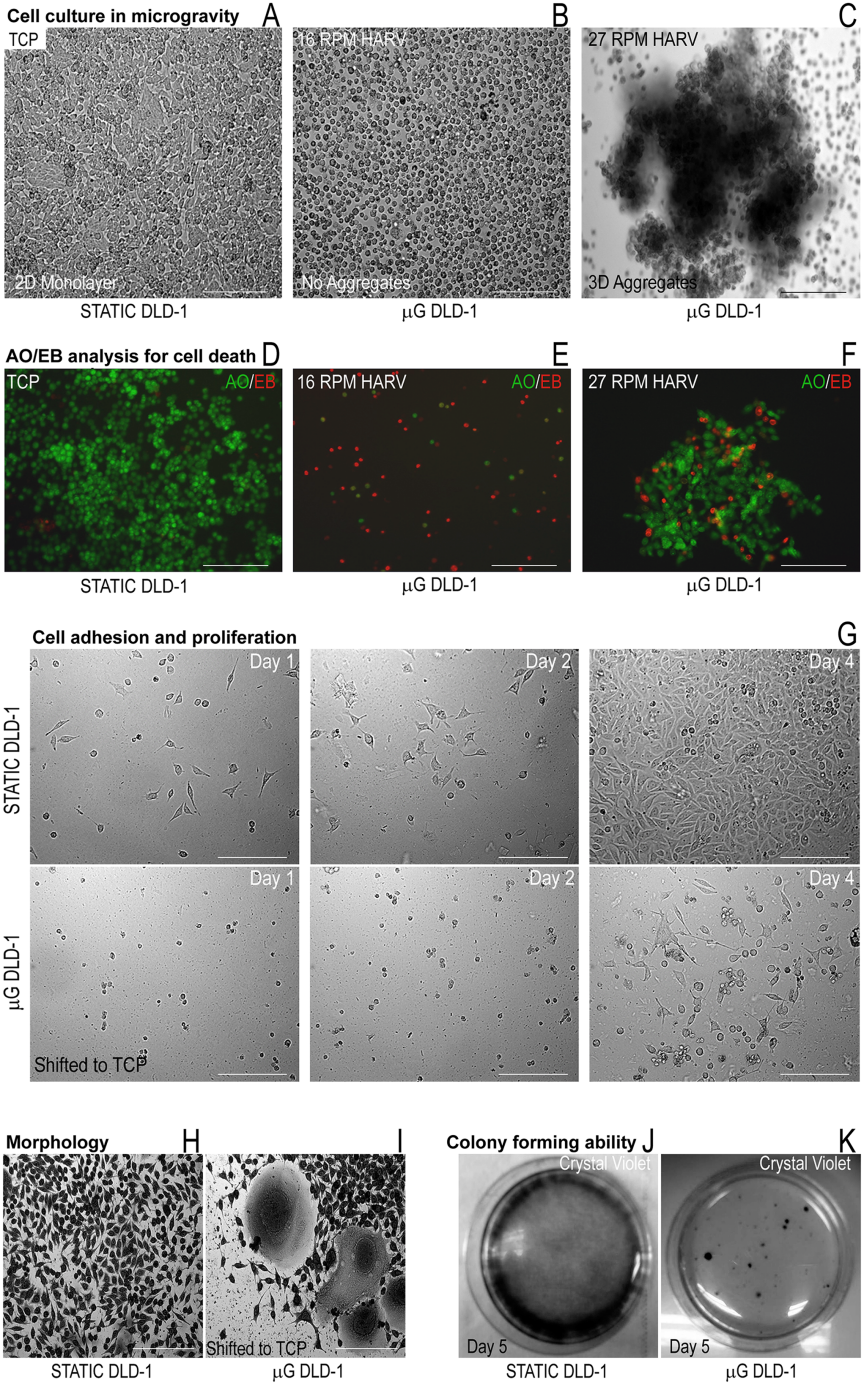
Effect of microgravity on cell morphology and cell viability of DLD-1 cell cultures. **A** DLD-1 cell cultures; Static culture (control) **B** DLD-1 Microgravity culture at 16 RPM **C** DLD-1 Microgravity culture at 27RPM **Differential staining to detect apoptotic population D** DLD-1Static monolayer cultures **E** Microgravity cultures of DLD1 at 16 RPM **F** Microgravity cultures of DLD1 at 27 RPM **G Cell adhesion and proliferation assay** Top panel—static cultures, bottom panel—microgravity cultures shifted to static TCP **H** Morphological changes in DLD-1; Crystal violet staining of DLD-1 cells in static monolayer culture **I** Crystal violet staining of DLD-1 cells after transfer of cell aggregates from microgravity to TCP **J** Colony forming ability assay; Static cultures **K** Colony forming ability assay; DLD-1 cells after transfer of cell aggregates from microgravity to TCP

**Fig 2:**
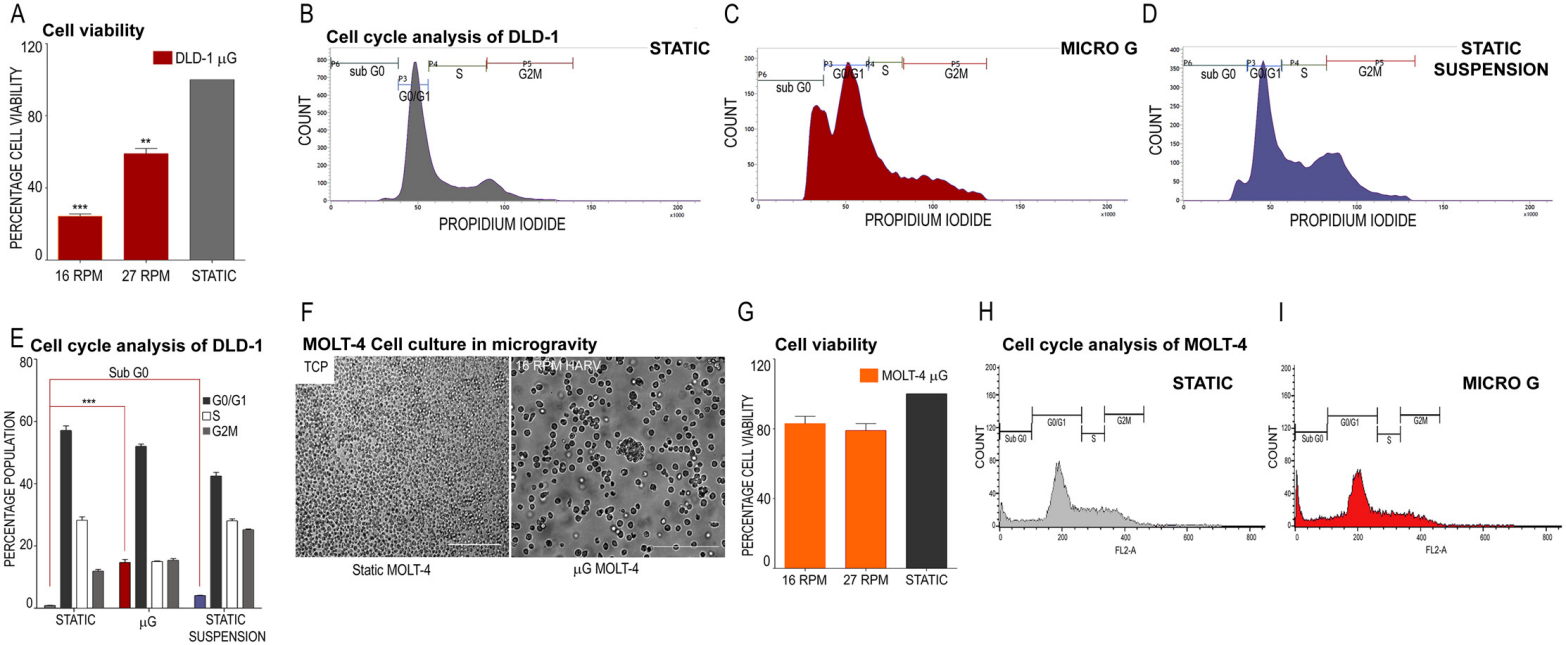
Effect of microgravity on cell viability and cell cycle of DLD-1 and MOLT-4 cell lines. **A** Cell viability assay for DLD-1 cells; Viability measured for microgravity cultures (16 RPM and 27 RPM) and static cultures using MTT **B** Cell cycle analysis for DLD-1 cells; Static **C** Cell cycle analysis; Microgravity **D** Cell cycle analysis; Static suspensions on agar underlays **E** The average sub G0 population in replicates of cell cycle analysis for microgravity, static and static suspension cultures of DLD-1 cells **F MOLT-4 cell culture** Static and Microgravity cultures of MOLT-4 **G Cell viability assay** Viability measured for microgravity cultures (16 RPM and 27 RPM) and static cultures using MTT **H** Cell cycle analysis; Static **I** Cell cycle analysis; Microgravity cultures of MOLT-4.

Availability of a surface to adhere for cells on the TCP is a stimulus for growth for adherent cells such as DLD-1 and therefore, static cultures in TCP were confluent (Fig 1A). Slow rotations per minute (RPM) of the HARV (16 RPM, Fig 1B) did not allow the cells to coalesce but cells could form aggregates at 27 RPM (Fig 1C). Staining with AO/EB demonstrated no cell death in static TCP cultures (Fig 1D) but revealed cell death in microgravity cultures of DLD1 at 16 RPM (Fig 1E). Cell viability was improved when cells aggregated at 27 RPM (Fig 1F). When these cell aggregates were enzymatically dissociated and cultured in a TCP after 48 hours in microgravity, cells took 48 hours to adhere (Fig 1G, Day 2) while cells from static cultures adhered within 24 hours (Fig 1G, Day 1) and produced confluent cultures by day 4. Microgravity has been shown to affect several cell functions [7] and the expression or functioning of cell adhesion molecules could be affected [8] reducing the number of DLD-1 cells that could adhere to the TCP. When such cell aggregates were plated in TCP without enzymatic dissociation, the morphology of DLD-1 cells was altered (Fig 1H and 1I). Crystal violet staining of cells grown in static monolayer culture shows the typical morphology of DLD-1 cells (Fig 1H) while cell aggregates assume a growth pattern similar to an explant culture and peripheral cells contained a large cytoplasm and nucleus (Fig 1I). The enlarged cell maybe due to the cytoskeletal changes during growth in microgravity [9] or cells, having lost control over the cell cycle, may accumulate pre-mitotic protein in the cytoplasm with polyploidy in the nucleus [9] increasing in size. A colony forming assay (CFA) on DLD-1 cultures in microgravity shifted to static TCP after enzymatic dissociation (Fig 1J and 1K) revealed the reduced potential of DLD-1 cells to form colonies (Fig 1K) as compared to static cells (Fig 1J). The reduced proliferative capacity of cultures in microgravity was confirmed by a cell viability assay using MTT. Static cultures were 41% more viable than 27 RPM cultures and 75% more viable than 16 RPM cultures in microgravity (Fig 2A). When these results are taken together, microgravity appears to significantly affect the viability and proliferation of DLD-1 cells. Flow cytometry analysis of PI loaded DLD-1 cells cultured under microgravity was compared to the cell cycle profile of cultures in static TCP and static suspension on agar underlay (Fig 2B, 2C and 2D). From lack of anchorage, static suspension cell cultures on agar also aggregate similar to the RCCS, however the simulation of microgravity is absent. DLD-1 cultures in microgravity had a substantial population of cells in the sub G0 phase although a large population of cells was still viable in microgravity (Fig 2C). Static monolayer, TCP cultures were completely viable (Fig 2B) and significantly, the static suspension cultures did not demonstrate a sub G0 phase and had profile similar to the static control (Fig 2D). Fig 2E shows the average sub G0 phase population across the three culture conditions in replicates of cell cycle analysis, which confirmed that reduced viability was an exclusive effect of microgravity in DLD-1 cell aggregates. The MOLT-4 cell line grows as a suspension culture and it did not aggregate favorably in microgravity at high or low RPMs, showing individual cells in the HARV (Fig 2F, 16 RPM HARV). However, cell viability was reduced by 20% as compared to the static control in both RPM at 48 hours (Fig 2G) which correlated with reduced cell numbers under microscopic observation after 48 hours (Fig 2F, 16 RPM HARV). Flow cytometry detected a small apoptotic population (Sub G0 stage) in cell cycle analysis of MOLT-4 cultures in microgravity at 16 RPM (Fig 2H and 2I).

### Real Time PCR for gene expression analysis

**Fig 3:**
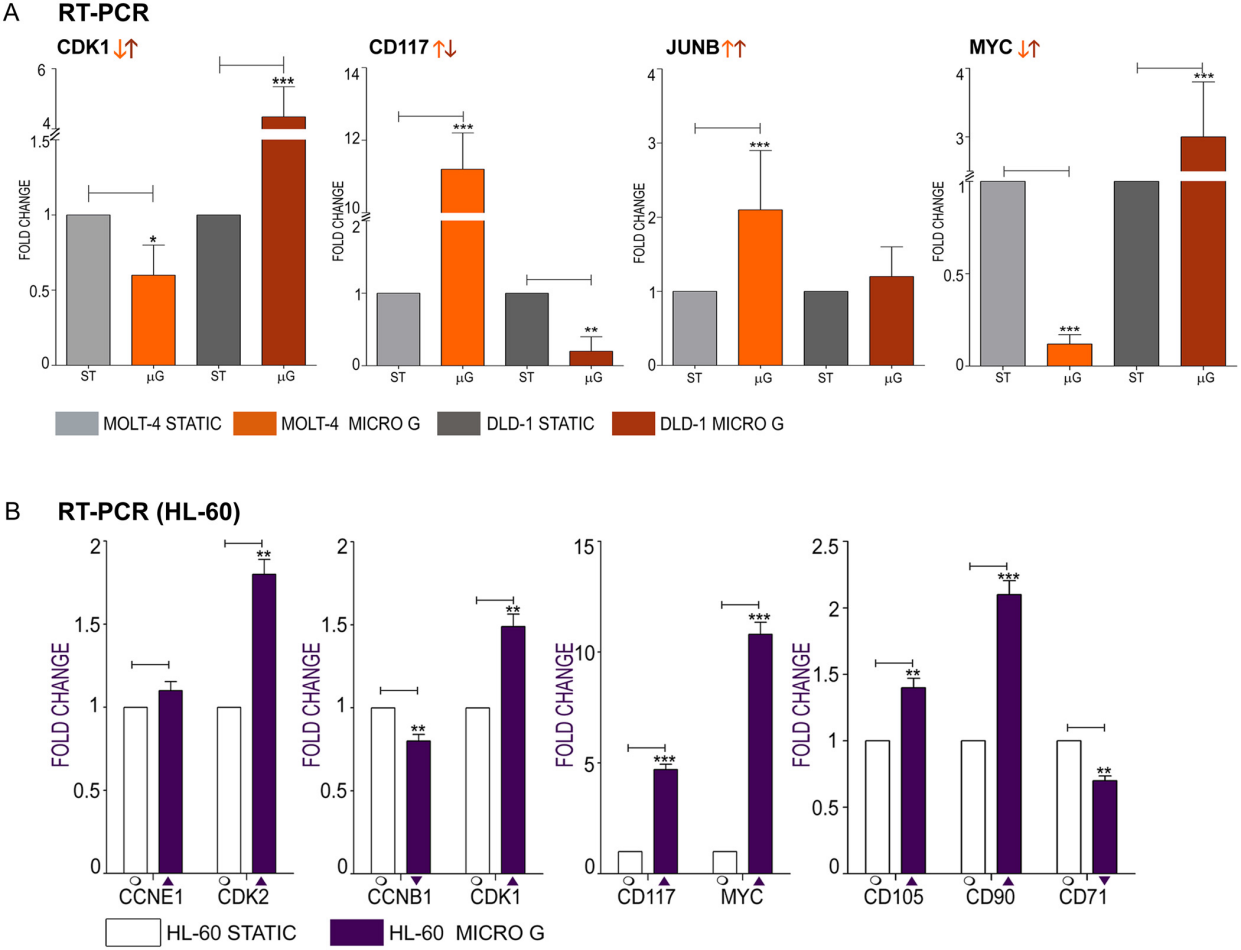
Quantitative PCR analysis for changes in mRNA expression of significant, candidate genes involved in cell proliferation and cancer. **A** *CDK1*—Cell cycle kinase gene, *CD117*—proto-oncogene, *JUNB*—transcription factor and immediate early gene, *MYC*—proto-oncogene expression in DLD-1 and MOLT-4 **B** Real time PCR analysis in HL-60 *CCNE1* and *CDK2*, *CCNB1* and *CDK1*, Oncogenes: *CD117* and *MYC*, Cancer prognostic markers *CD105*, *CD90* and *CD71*.

To affect central processes of cancer such as cell proliferation and cell cycle, microgravity must significantly influence fundamental functions of the cell such as gene expression. We measured the mRNA levels of significant genes involved in cell cycle and cancer progression to check for their dysregulation under microgravity. Cyclin gene expression levels significantly influence cancer progression and metastasis as they can direct cell proliferation or apoptosis. Cdk are essential for G1/S and G2/M phase transitions of the cell cycle and their dysregulated gene expression can affect the progression of the cell cycle. The transcription of *CDK1* is regulated such that it functions during the mitotic prophase and metaphase [10]. *CDK1* expression was down regulated in MOLT-4 and upregulated in DLD-1 (5-fold over static control) (Fig 3A). The expression of genes fundamental to cancer development and progression, which include oncogenes and potential cancer stem cell markers, were dysregulated in microgravity. *CD117* (receptor tyrosine kinase—c-kit) expression was upregulated by 11.2 fold in MOLT-4 and downregulated by 0.2 fold in DLD-1 under microgravity (Fig 3A). High c-kit expression protects colon carcinoma cells against apoptosis and enhances their invasive potential [11]; therefore, c-kit downregulation in DLD-1 under microgravity may be significant. DLD-1 constitutively over expresses the *MYC* gene [12] under normal conditions. Overexpression of *MYC* sensitizes cells to apoptosis and under microgravity *MYC* gene expression was further increased in DLD-1 by 3 fold (Fig 3A). MOLT4 expressed lowered levels of *MYC* (0.4 fold) in microgravity (Fig 3A). *JUNB* encodes a transcriptional regulator of cell proliferation genes and is part of the immediate early gene family [13]. One of the most significant genes to be dysregulated in both cell lines in microgravity, *JUNB* is upregulated in microgravity by 2.1 and 1.2 fold in MOLT-4 and DLD-1 respectively (Fig 3A).

### Gene expression analysis in HL-60, a promyelocytic leukemia cell line

As an additional control for blood tumor (and suspension) cultures, we checked the gene expression levels of the cell cycle genes and oncogenes in a promyelocytic leukemia cell line, HL-60. Real time PCR revealed the up regulation of *CCNE1* and *CDK2* in HARV cultures with *CDK2* being significantly up-regulated (1.1 and 1.8 fold respectively) (Fig 3B). *CCNB1* and *CDK1* gene expression was dysregulated with *CDK1* being up regulated 1.5 fold and *CCNB1* down regulated by 0.8 fold (Fig 3B). Significantly, the proto oncogenes *CD117* and *MYC* were highly up regulated in microgravity by 4.7 fold and 10.8 fold respectively (Fig 3B). Similar to the DLD-1 cell line, HL-60 also over expresses the *MYC* gene constitutively under standard conditions. The prognostic markers *CD71*, *CD105* and *CD90* were dysregulated under microgravity by 0.75 fold (downregulated), 1.4 fold (upregulated) and 2.1 fold (upregulated) respectively (Fig 3B). Endoglin (*CD105)* aids neovascularization in cancer [14] and *CD90* expression indicates a positive prognosis as leukemic progenitor cells in AML that are capable of maintaining the disease in vitro and in vivo do not express CD90 [15]. Real time PCR analysis of a candidate cell cycle, oncogene, transcription factor and cancer progression marker showed both upregulation and downregulation. As the cell cycle is regulated by a multitude of factors many of which could be affected by microgravity, a ‘collective’ downregulation or dysregulation of processes associated with cell cycle could validate the observed physiological halt or reduction in cell proliferation under microgravity. Towards this aim, a genome wide expression profiling using DNA microarray was carried out. The genomic profiling also allowed us to speculate on the effect of microgravity on central pathways in cancer such as the Notch signaling system, and expression levels of novel regulators such as microRNA.

### Microarray analysis of DLD-1 and MOLT-4 cells cultured in microgravity

Microarray analysis revealed 1801 and 2542 genes up and down regulated more than 2 fold in DLD-1 cells cultured in microgravity compared to static control. MOLT-4 cultures under microgravity differentially expressed a total of 349 and 444 genes up and down regulated over 2 fold, respectively. Table 1 represents a short list of common genes deregulated among both cell lines. A complete list of highly deregulated genes among both cell lines is provided in S2, S3 and S4 Tables. The highly dysregulated genes represented in the supporting information tables contain interesting candidate genes such as the ribonucleotide reductase M2 (*RRM2*) subunit which is the most down regulated gene in DLD-1 under microgravity. RRM2 overexpression may be associated with Colo Rectal cancer (CRC) progression and may play an important role in the infiltration and metastasis of CRC [16]. It serves as a prognostic biomarker and predicts poor survival of colorectal cancers [17]. While both cell lines exhibited changes in cell cycle and cell viability, microgravity elicited a greater response from the solid tumor cell line DLD-1. Sub lethal stress can push a cell into a state that is similar to replicative senescence [18]. Stress-induced premature senescence (SIPS) can occur after DNA damage, oxidative stress and treatment with histone deacetylase inhibitors [18]. The phenomenon of SIPS can explain the loss in cell viability in both cell lines. Microarray analysis revealed the downregulation of the retinoblastoma gene (*RB1*; -0.42 log fold change) in DLD-1 cells under microgravity and as the presence of the RB1 protein is necessary for SIPS [19], the response of DLD-1 cells to microgravity may not be through SIPS and its related pathways. Conversely MOLT-4 cells show upregulated RB1 expression (0.47 log fold change) under microgravity and the loss in cell viability may be attributed to SIPS. This could also explain the relatively lesser number of genes that were differentially expressed in MOLT-4 cells compared to the gene expression profile of DLD-1 cells under microgravity. Other biomarkers of SIPS, apolipoprotein J and fibronectin, which are overexpressed in replicative senescence and SIPS [19], were not differentially expressed in DLD-1 or MOLT-4 under microgravity. The mechanism of SIPS is not clearly understood as yet and whether microgravity can be a trigger for SIPS pathways needs to be confirmed. The data discussed in this publication have been deposited in NCBI's Gene Expression Omnibus and are accessible through GEO Series accession number GSE69271 (http://www.ncbi.nlm.nih.gov/geo/query/acc.cgi?acc=GSE69271). The differentially expressed genes from the microarray data were studied for their overabundance in different Gene Ontology (GO) terms as well as pathways using the microarray analysis software DAVID. The enrichment score of individual GO terms that the genes associated with was used to identify processes that were significantly dysregulated. GO or functional enrichment analysis was performed with over 2 fold-differentially expressed genes in both cell lines. Functional classification of genes based on similarity in function or family was carried out.

**Table 1:** Commonly deregulated genes in DLD-1 and MOLT-4 cells under microgravity.

**UPREGULATED GENES**			
**GENE SYMBOL**	**GENE NAME**	**Log FC DLD-1**	**Log FC MOLT-4**
**ARRDC3**	arrestin domain containing 3	3.6286426	2.5424619
**ATF3**	activating transcription factor 3	2.6872826	1.0043197
**CCPG1**	cell cycle progression 1	1.5802538	1.8111625
**CDKN2AIP**	CDKN2A interacting protein	1.6052608	1.0670295
**CDKN2D**	cyclin-dependent kinase inhibitor 2D (p19, inhibits CDK4)	1.3287859	1.0733795
**CREBBP**	CREB binding protein	1.6003945	1.1786065
**CREBRF**	CREB3 regulatory factor	1.7348738	1.2481394
**CXCL3**	chemokine (C-X-C motif) ligand 3	1.6632779	1.1129959
**DDIT3**	DNA-damage-inducible transcript 3	2.0701203	1.280817
**EGR2**	early growth response 2	1.3966465	3.9244506
**ETS1**	v-ets erythroblastosis virus E26 oncogene homolog 1 (avian)	1.3279192	1.0439773
**ETV5**	ets variant 5	1.2021747	2.488372
**FGF7**	fibroblast growth factor 7	2.2669797	1.1106136
**GORAB**	golgin, RAB6-interacting	1.1204505	1.0041857
**HDAC9**	histone deacetylase 9	1.0828898	1.4470437
**HINT3**	histidine triad nucleotide binding protein 3	1.7376733	1.7083709
**HIVEP2**	human immunodeficiency virus type I enhancer binding protein 2	1.2518463	1.2009206
**IRS2**	insulin receptor substrate 2	2.5675535	1.0355048
**JUN**	jun proto-oncogene	2.8459454	3.4484391
**MIR1304**	microRNA 1304	1.694859	1.0175457
**NCOA7**	nuclear receptor coactivator 7	1.3721471	1.0397696
**NDFIP2**	Nedd4 family interacting protein 2	1.0812235	1.1697233
**PIBF1**	progesterone immunomodulatory binding factor 1	1.6272297	1.2976668
**PLEKHF2**	pleckstrin homology domain containing, family F (with FYVE domain)	2.5150435	1.105514
**PTEN**	phosphatase and tensin homolog	1.2177935	1.0146773
**RAB30**	RAB30, member RAS oncogene family	1.8227141	1.6890364
**SKIL**	SKI-like oncogene	1.7823422	1.4903564
**SMAD7**	SMAD family member 7	1.0359683	1.338062
**TNFAIP3**	tumor necrosis factor, alpha-induced protein 3	1.2988296	1.3440051
**XIAP**	X-linked inhibitor of apoptosis	1.3545609	1.2860351
**ZFAND2A**	zinc finger, AN1-type domain 2A	1.9506464	1.2355728
**ZFY**	zinc finger protein, Y-linked	2.238248	1.2044845
**ZMYM5**	zinc finger, MYM-type 5	1.7800057	1.4686751
**DOWNREGULATED GENES**			
**GENE SYMBOL**	**GENE NAME**	**Log FC DLD-1**	**Log FC MOLT-4**
**ASIC1**	acid-sensing (proton-gated) ion channel 1	-1.1945584	-1.4278164
**CD24**	CD24 molecule	-2.1321063	-1.3863628
**CDCA7L**	cell division cycle associated 7-like	-1.6832285	-1.0647273
**CDKN1C**	cyclin-dependent kinase inhibitor 1C (p57, Kip2)	-1.2500896	-1.0963018
**DHFR**	dihydrofolate reductase	-1.8050942	-1.0276904
**DNHD1**	dynein heavy chain domain 1	-1.464865	-1.0485542
**DUT**	deoxyuridine triphosphatase	-1.0620422	-1.175415
**EEF1A1**	eukaryotic translation elongation factor 1 alpha 1	-1.0099685	-1.0678835
**EIF4A1**	eukaryotic translation initiation factor 4A1	-1.0106502	-1.0274181
**ENSA**	endosulfine alpha	-1.0821726	-1.1114485
**FANCL**	Fanconi anemia, complementation group L	-1.2304835	-1.0152798
**FAR1**	fatty acyl CoA reductase 1	-1.0487332	-1.0339952
**FGFR3**	fibroblast growth factor receptor 3	-1.6760249	-1.3410914
**GSPT1**	G1 to S phase transition 1	-1.3995273	-1.0519781
**GSTA4**	glutathione S-transferase alpha 4	-1.1619577	-1.0276983
**HES4**	hairy and enhancer of split 4 (Drosophila)	-1.1826415	-2.0193136
**HMGB1**	high mobility group box 1	-1.002748	-1.2596858
**HMGB3**	high mobility group box 3	-1.2634602	-1.1885982
**HSPA4**	heat shock 70kDa protein 4	-1.5835454	-1.2644181
**IFI30**	interferon, gamma-inducible protein 30	-1.5613956	-1.5471194
**IFRD2**	interferon-related developmental regulator 2	-1.1622665	-1.0979714
**JPH1**	junctophilin 1	-1.1266716	-1.6309524
**LOC100653301 /// NRBP2**	nuclear receptor-binding protein 2-like /// nuclear receptor binding protein 2	-1.5101182	-1.0871661
**MTPAP**	mitochondrial poly(A) polymerase	-2.2048273	-1.046771
**NEURL1B**	neuralized homolog 1B (Drosophila)	-1.2790649	-1.0592682
**NFIA**	nuclear factor I/A	-1.2247176	-1.1426408
**PARP1**	poly (ADP-ribose) polymerase 1	-1.6084354	-1.0683279
**PHKA1**	phosphorylase kinase, alpha 1 (muscle)	-1.4648097	-1.3255553
**PLXNA1**	plexin A1	-1.1962962	-1.3295693
**PNPT1**	polyribonucleotide nucleotidyltransferase 1	-1.3692455	-1.0695791
**POLR3H**	polymerase (RNA) III (DNA directed) polypeptide H (22.9kD)	-1.0398519	-1.1780653
**RBBP4**	retinoblastoma binding protein 4	-1.0829744	-1.1924767
**TUBB**	tubulin, beta class I	-1.1920624	-1.0390439

Table shows a section of commonly deregulated genes under microgravity in both cell lines as revealed by microarray analysis. The complete list of genes is provided in S5 Table.

### Validation of candidate genes selected from microarray data

**Fig 4:**
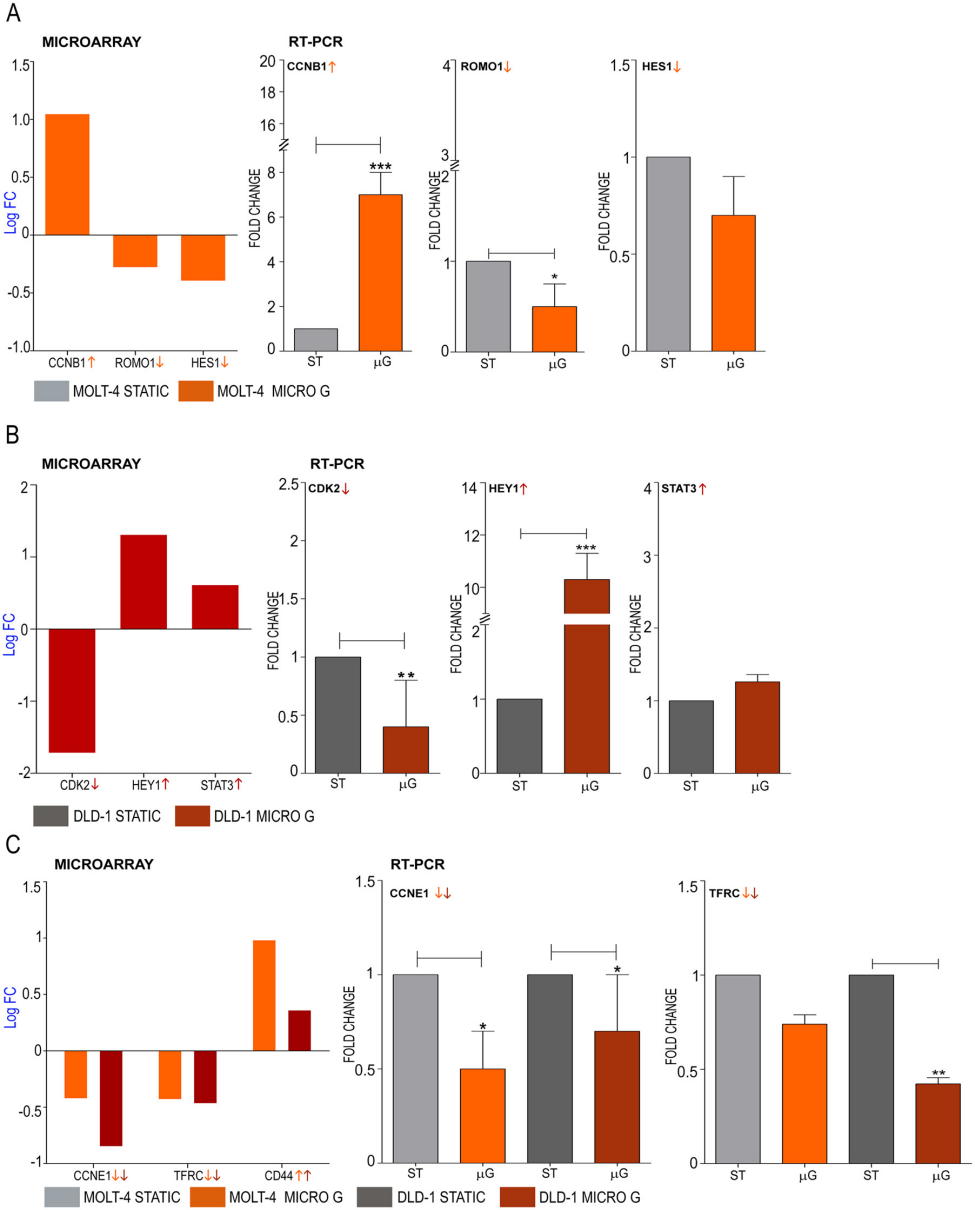
Effect of microgravity on gene expression levels in DLD-1 and MOLT-4 cell cultures; Validation of microarray analysis by real time PCR and western blotting. **A** Log fold change of *CCNB1*, *ROMO1* and *HES1* deregulation in MOLT-4 cells under microgravity as observed by microarray analysis validated by real time PCR **B** Log fold change of *CDK2*, *HEY1* and *STAT3* deregulation in DLD-1 cells under microgravity as observed by microarray analysis validated by real time PCR **C** Log fold change of commonly up and downregulated genes *CCNE1*, *TFRC (CD71)* and *CD44* as observed by microarray analysis validated by real time PCR for*CCNE1 and TFRC (CD71)*.

**Fig 5:**
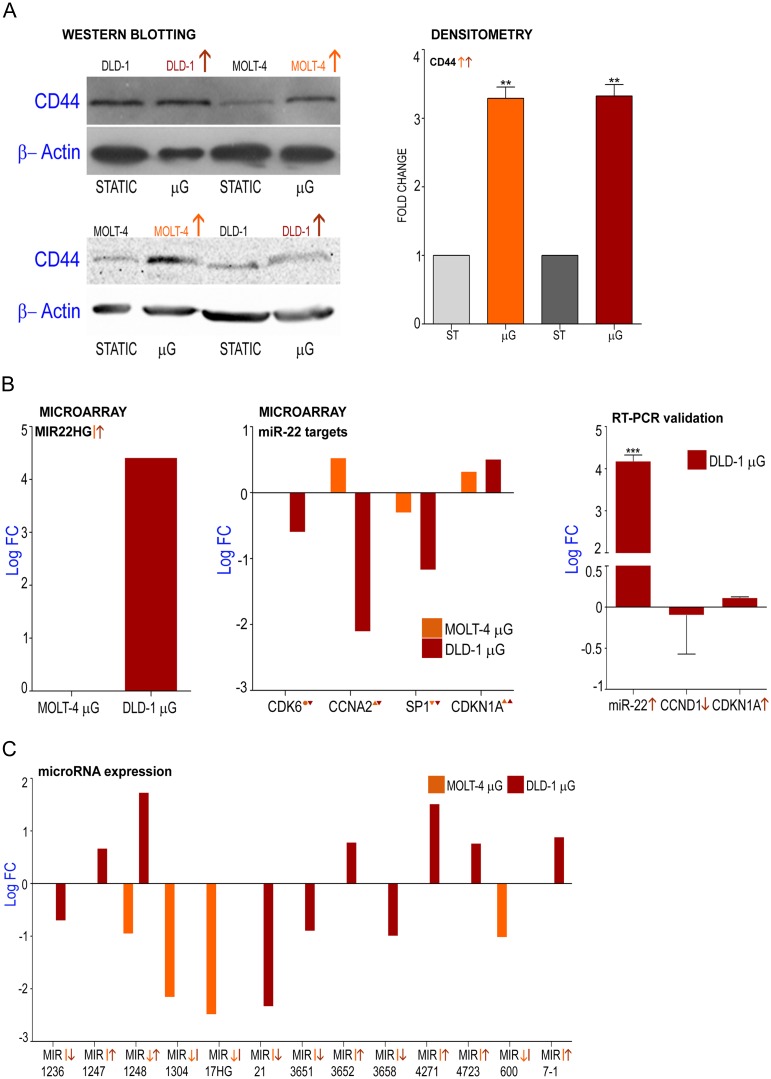
Dysregulation of stem cell marker CD44 and tumor suppressor microRNA under microgravity. **A** Validation of microarray data for *CD44* by Western blotting for CD44 protein and beta-actin in static and Microgravity (μG) cultures of DLD1 and MOLT-4 and Densitometric analysis of western blots. **B** MIR22HG expression under microgravity; Overexpressed MIR22HG, host gene of miR-22 microRNA in DLD-1 cells under microgravity, MOLT-4 cells show no differential expression; Levels of dysregulation of direct targets of miR-22 microRNA *CDK6*, *CCNA2*, *SP1* and *CDKN1A* in microarray data; RT-PCR validation of microRNA miR-22 levels and target genes in DLD-1 shows over expressed microRNA miR-22 in DLD-1 cells under microgravity confirming upregulation in microarray data. No significant dysregulation of direct targets *CDKN1A* (similar to expression levels in microarray data) and *CCND1A* **C** GO analysis (by DAVID) of microarray data to depict other dysregulated microRNA host genes in DLD-1 and MOLT-4 cells under microgravity.

Microarray analysis revealed the upregulation of *CCNB1* and the down regulation of *ROMO1* and *HES1* genes when MOLT-4 cells were cultured under microgravity (Fig 4A). Similarly, microarray analysis demonstrated the down regulation of *CDK2* gene and the upregulation of *STAT3* and *HEY1* genes in DLD-1 cells cultured under microgravity (Fig 4B). Microarray analysis revealed that both cell lines commonly showed the down regulation of *CCNE1* and the upregulation of *CD71* and *CD44* genes (Fig 4C, Fig 5A). Significantly, the deregulation of the microRNA-22 host gene and its targets was also under microgravity (Fig 5B). mRNA expression of these candidate genes representative of the cell cycle, transcriptional regulation and cancer progression were validated by quantitative real time PCR. Cyclin B1 which has been reported as constitutively overexpressed in human colorectal cancers [20] is over expressed in MOLT-4 cells which showed a 7.5 fold increase of *CCNB1* mRNA expression under microgravity (Fig 4A). Lowered expression of *ROMO1* leads to inhibition of cell growth [21] and MOLT-4 cells expressed 0.5 fold less *ROMO1* than the static control (Fig 4A). Transcriptional and replication controllers regulate oncogenes and prognostic markers and influence cell cycle events. *HES1* is a transcriptional repressor and is involved in DNA repair [22] and *HES1* gene expression is controlled by the Notch and Jun signaling system [23]. *HES1* gene expression is down regulated by 0.7 fold in MOLT-4 (Fig 4A) under microgravity. *CDK2* gene expression in DLD-1 cells was 0.5 fold lower compared to static control (Fig 4B) while *HEY1*, a transcriptional regulator was highly up regulated by 10.3 fold (Fig 4B). Signal transducer and activator of transcription 3 (*STAT3*) is an oncogenic transcription factor which is activated and aberrantly expressed in many colorectal cancers [24] and the genes upregulation is validated by RT-PCR (Fig 4B). Cyclin E1 gene expression is down regulated in both cell lines under microgravity (Fig 4C). Cyclin E1 controls the progression of the cell cycle through the G1 phase by its interaction with cyclin dependent kinase 2 [25]. Similarly, both cell lines expressed lowered levels of *CD71*, which encodes a transmembrane glycoprotein that is responsible for cellular iron uptake (Fig 4C). DLD-1 expressed significantly lower levels (0.42 Fold) in microgravity. Higher expression of *CD71* is associated with negative prognosis for many solid tumors and some lymphomas [26,27] and numerous studies have found a positive correlation between iron storage and the risk of tumors such as in colorectal carcinoma [28]. Significantly, the *CD44* gene was upregulated in both cell lines (Fig 4C, Fig 5A). While most isoforms of *CD44* are associated with the malignant form of the disease, some forms of *CD44* prevent the tumor cells from spreading out of the primary site [29]. As *CD44* is expressed in both colon and lymphoid cancers and as mRNA analysis would involve multiple variants, we investigated its protein levels in DLD-1 and MOLT-4 cultures in microgravity. Western blotting for standard isoform of *CD44* shows higher levels of the protein in both cell lines over static control (Fig 5A). Densitometric analysis of the bands and normalization with β-actin values demonstrates significant up regulation of *CD44* protein in microgravity. microRNA have been identified as potential oncogenes or tumor suppressors [30]. The miR-22 host gene, *MIR22HG* was highly upregulated in DLD-1 (Log fold 4.4) but not differentially expressed in MOLT-4 (Fig 5B). miR-22 functions as a tumor suppressor through post-transcriptional regulation of p21 to determine cell fate [31]. It represses cancer progression by inducing cellular senescence [32] and controls EVI-1 oncogene expression in metastatic breast cancer cells [33]. Some targets of miR-22 such as *SP1*, *CDK6* and *CCNA2* were also significantly downregulated (Fig 5B) while others such as p21 (*CDKN1A*) were not significantly dysregulated. The farnesoid X receptor regulates miR-22 which targets *CCNA2* in colon and liver cancer cells [34]. Real time PCR for miR-22 microRNA also showed a 4.18 log fold upregulation in DLD-1 cells under microgravity (Fig 5B) confirming the fold change observed in microarray analysis. Real Time PCR for miR-22 targets—*CCND1* and *CDKN1A* however, did not show significant dysregulation with -0.09 log fold (down regulation) and 0.11 log fold (up regulation) change, respectively (Fig 5B). Other micro RNA host genes including *MIR17HG*, was significantly downregulated in MOLT-4 while not differentially expressed in DLD-1 (Fig 5B). The *MIR21HG* is significantly downregulated in DLD-1 while it is not differentially expressed in MOLT-4 (Fig 5B). The *MIR17HG* or MiR-17-92 Cluster Host Gene encodes for six miRNAs that influence cell survival, proliferation, differentiation, and angiogenesis [35]. miR-21 downregulates tumor suppressor Pdcd4 and stimulates invasion, intravasation and metastasis in colorectal cancer [36]. The downregulation of miR-21 also induces differentiation of chemoresistant colon cancer cells enhancing their susceptibility to therapy [37].

### Dysregulation of genes involved with the Notch signaling system and microRNA processing

**Fig 6:**
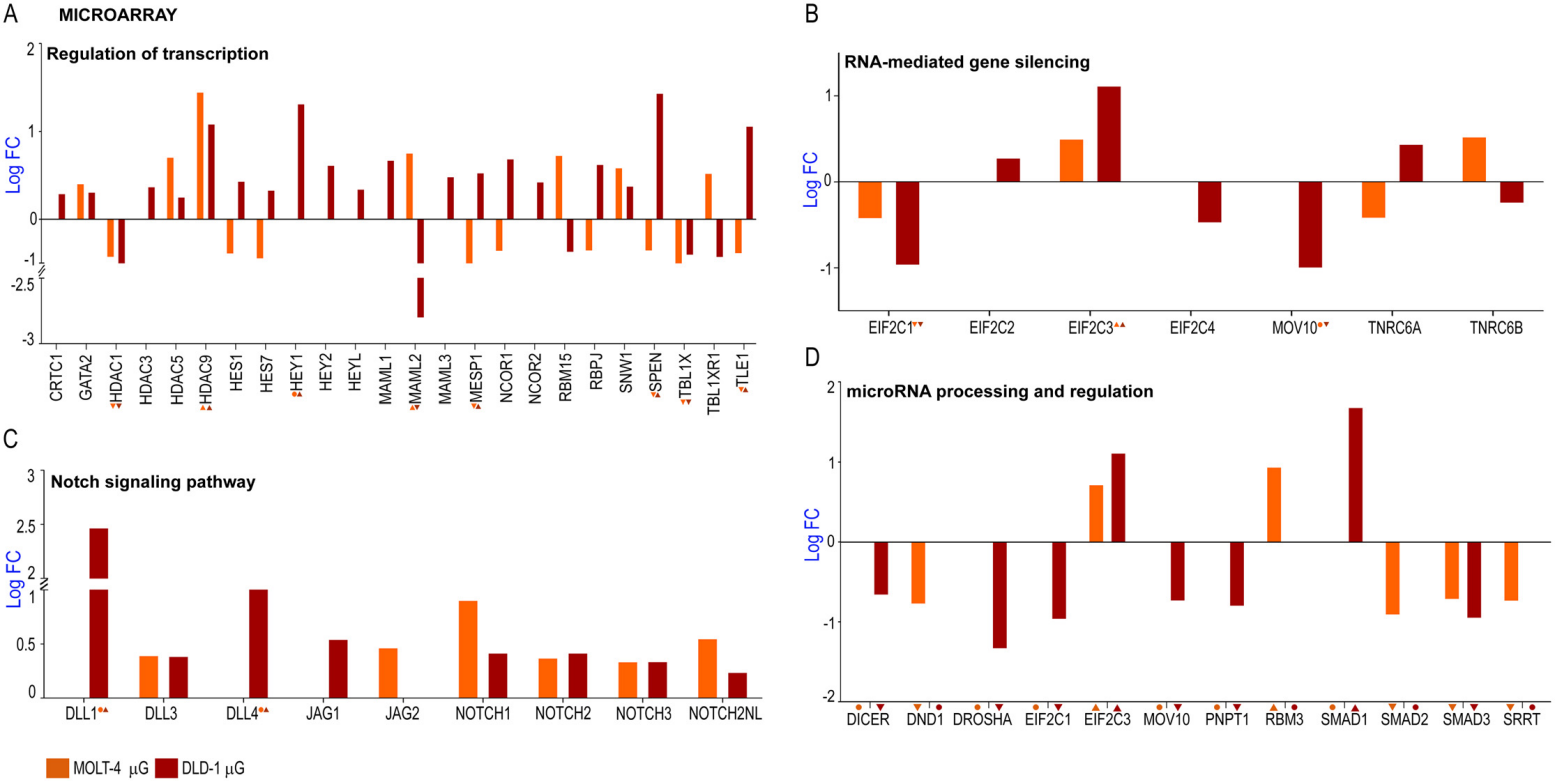
Analysis of microarray data using Gene Functional Classification and Functional Annotation; Dysregulation of genes involved in the Notch signaling system and microRNA processing and regulation. **A** Regulation of transcription **B** RNA mediated gene silencing (PTGS) **C** Notch signaling pathway **D** Dysregulated microRNA processors and regulators.

The Notch signaling system plays an important role in the mechanical unloading of bone in microgravity, and changes to the mesenchymal and hematopoietic stem cell compartments. The Notch pathway is also significant in cancer progression and microRNA processing. As one of the highly deregulated genes observed in the microarray analysis is a tumor suppressor microRNA, we looked at other genes and pathways in the Notch signaling system that could be deregulated. GO analysis revealed multiple genes that were functionally clustered under processes associated with the Notch system (Fig 6A, 6B and 6C). Significantly dysregulated genes involved in transcriptional regulation include *HDAC1*, *HDAC3*, *HEY1*, *MAML2*, *MESP1*, *SPEN*, *TBL1X and TLE1* (Fig 6A). The *HDAC1* and *HDAC3* genes are potential tumor suppressors that interact with retinoblastoma 1 and p53 proteins respectively [38] while *MAML2* is a potential oncogene [39] that is down regulated in DLD-1 by more than 2 log fold and upregulated in MOLT-4 (Fig 6A). Major genes involved in post transcriptional gene silencing were dysregulated under microgravity (Fig 6B) such as *EIF2C2* which was dysregulated only in DLD-1 while *EIF2C1* and *EIF2C3* were significantly downregulated and upregulated in both cell lines (Fig 6B). The human eukaryotic initiation factor 2C1 (*EIF2C1*) and *EIF2C2* are components of the core RNA-induced silencing complex (RISC) and are members of the argonaute protein family [40] that are potential biomarkers for human colon cancer [41]. DLL1 was significantly up regulated in the process of Notch signaling pathway by 2.46 log fold among other genes in DLD-1 (Fig 6C) but the gene was not differentially expressed in MOLT-4. *DLL4* and *JAG1* were differentially expressed in DLD-1 while *JAG2* was expressed only in MOLT-4 (Fig 6C). Epigenetic regulation of DLL1 controls Notch1 activation in gastric cancer and along with *DLL4*; *DLL1* is required for homeostasis of intestinal stem cells [42,43]. Significantly, all the NOTCH genes were up regulated in both cell lines (Fig 6C) and these genes encode receptors for membrane-bound ligands Jagged1/2 and Delta1. *JAG1* is involved in cell-fate decisions during hematopoiesis while *JAG2* is overexpressed in malignant myeloma plasma cells [44,45]. *TLE1*, *TLE2* and *FBXW7* genes, which have the conserved WD40 repeat domain, were dysregulated (S1A Fig) in both cell lines while *TLE3* is highly upregulated in DLD-1 and not differentially expressed in MOLT-4. *FBXW7* is a potential tumor suppressor gene [46] involved in ubiquitination and subsequent degradation of cyclin E and MYC. The ribosomal Protein S27a (*RPS27A*) promotes proliferation, regulates cell cycle progression and inhibits apoptosis of leukemia cells. *RPS27A* was significantly dysregulated only in DLD-1 and not in MOLT-4 (S1B Fig). *APH1B* and the presenilin protein encoding genes, *PSEN2* and *PSENEN* were dysregulated only in the DLD-1 cell line (S1B Fig). *APH1B* encodes a subunit of the gammasecretase complex that catalyzes the cleavage of proteins such as Notch receptors and APP (beta-amyloid precursor protein) while *PSEN2* and *PSENEN* regulate APP processing through gamma-secretase [46] and are involved in Alzheimer’s disease [47]. A substantial number of genes involved in microRNA processing and regulation were dysregulated (Fig 6D) such as *DROSHA*, which is the core nuclease that executes the initiation step of miRNA processing in the nucleus [48] and *DICER*, the endoribonuclease that cleaves naturally occurring long dsRNAs and short hairpin pre-microRNAs (miRNA) into short interfering RNAs (siRNA) and mature microRNAs [49]. The SMAD proteins control DROSHA-mediated microRNA maturation [50] and the *SMAD1* gene is upregulated in DLD-1 while *SMAD2* is downregulated in MOLT-4 (Fig 6D). *SMAD3* is downregulated in both cell lines (Fig 6D). Micro RNA host genes including *MIR17HG*, was significantly downregulated in MOLT-4 while not differentially expressed in DLD-1 (Fig 6D). The *MIR21HG* is significantly downregulated in DLD-1 while it is not differentially expressed in MOLT-4 (Fig 6D). The *MIR17HG* or MiR-17-92 Cluster Host Gene encodes for six miRNAs that influence cell survival, proliferation, differentiation, and angiogenesis [35]. miR-21, miR-17 and miR-19a are directly involved in the proliferation and metastasis of colon cancer [51]. miR-21 downregulates tumor suppressor Pdcd4 and stimulates invasion, intravasation and metastasis in colorectal cancer [36]. The downregulation of miR-21 also induces differentiation of chemoresistant colon cancer cells enhancing their susceptibility to therapy [37].

### Overview of cellular processes and functions with deregulated genes under microgravity

**Fig 7:**
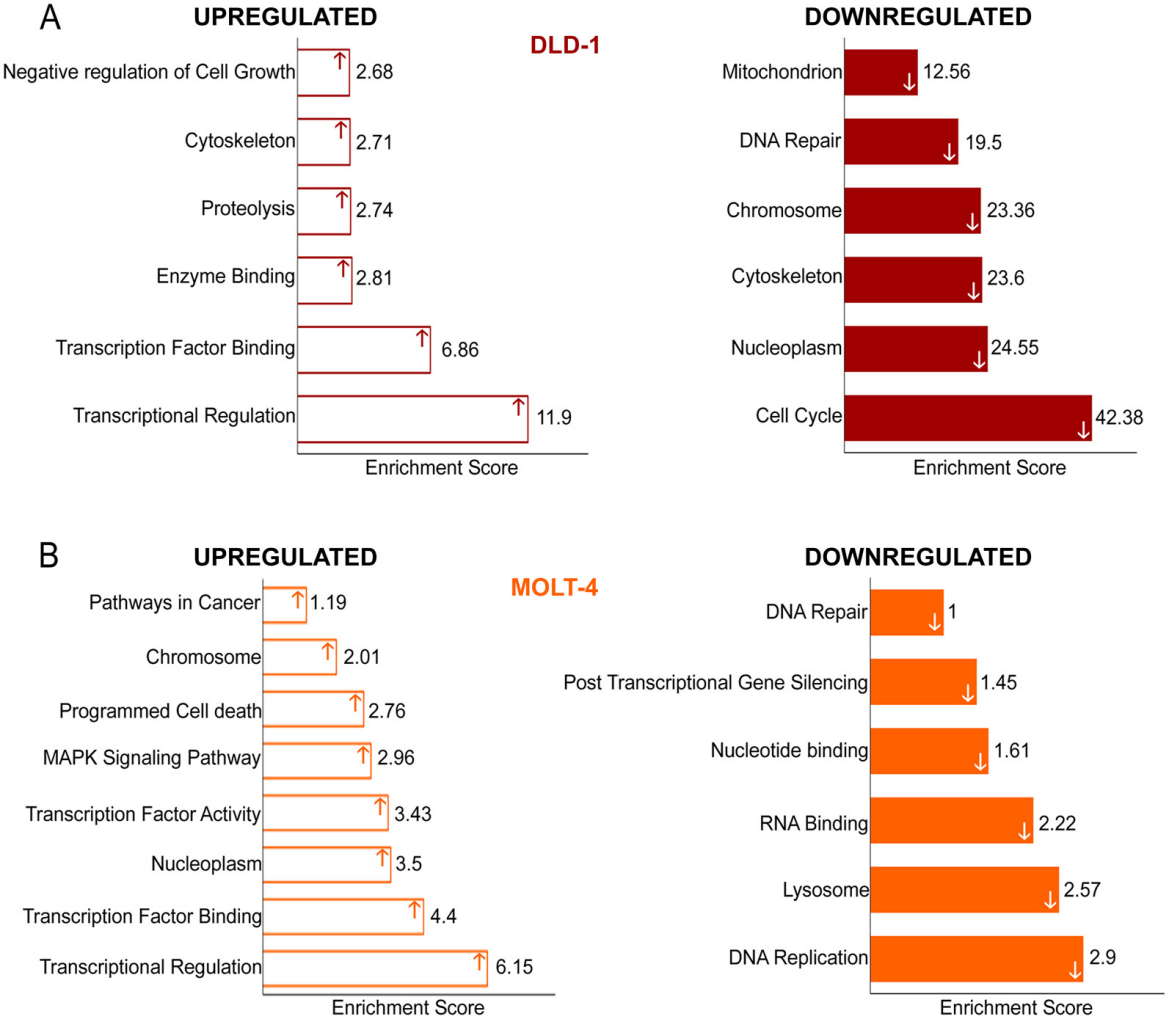
Functional annotation of microarray data using DAVID. **A** Functional annotation of upregulated and downregulated genes of DLD-1 cells under microgravity **B** Functional annotation of upregulated and downregulated genes of MOLT-4 cells under microgravity.

Fig 7 shows a summary view of the functional annotation clustering of GO terms using DAVID, demonstrating processes or functions which the highly dysregulated genes (> 2 log fold) in the DNA microarray list associated with. Upregulated genes were involved in functions associated with transcriptional regulation, proteolysis and negative regulation of cell growth (Fig 7A). Prominently, correlating with the observations in gene expression analysis by real time PCR and experimental procedures on the cell cycle progression of DLD-1 cells, the most significant cluster of genes with the highest enrichment score were involved in cell cycle (Fig 7A). All genes that were associated with these GO terms were downregulated. Other clusters that were associated with downregulated genes included cytoskeleton, nucleoplasm and DNA repair; biological processes that are vital to cell cycle and proliferation. MOLT-4 cells showed similar results with lower enrichment scores. Upregulated genes again clustered in transcriptional regulation and programmed cell death (Fig 7B) and significantly, in pathways in cancer. DNA replication was an enriched GO term in the downregulated gene cluster with many associated processes such as DNA repair and nucleotide binding also being enriched. From the functional annotation clustering of the deregulated list of genes, it can be surmised that microgravity has a suppressive effect on the progression of cancer, particularly in the colorectal cancer cell line- DLD-1.

### Conclusion

Although the mechanism of action is still unclear, normal cells, stem cells and cancer cells have altered physiological properties under microgravity. The cytoskeleton and the plasma membrane of the cell may be sensitive to changes in gravity [52] and respond by altering vital intracellular signaling networks or the modulation of proto oncogene expression through intracellular signaling pathways could be a mechanism of action [53]. It is of significance that cancer cell proliferation and progression can be altered by microgravity in DLD-1 cells and to a lesser extent, in MOLT-4 cells, as demonstrated by this study. Simulated microgravity may affect solid tumor cell lines markedly such as DLD-1, which showed a higher percentage of dysregulated genes compared to the hematological tumor cell line, MOLT-4. The process of cell cycle in DLD-1 cells was markedly affected with reduced viability, reduced colony forming ability, an apoptotic population and dysregulation in cell cycle genes. Real time PCR and western blotting also demonstrated dysregulation of significant oncogenes and cancer progression markers such as *JUNB*, *CD44*, *MYC* and *CD117*. This was corroborated with the downregulation of the process of cell cycle as demonstrated by the functional clustering of DNA microarray data using GO terms by DAVID. This study also demonstrated for the first time, the dysregulation of the microRNA host genome, miR-22 in a colorectal cancer cell line, DLD-1. Due to the significant tumor suppressive role of microRNA-22, its upregulation under microgravity may contribute to the anti-proliferative effect of microgravity. Identifying mechanisms by which microgravity influences miR-22 expression and the other dysregulated microRNA host genes identified in this study, may provide potential candidates for cancer therapy.

## Supporting Information

S1 Fig Analysis of microarray data using Gene Functional Classification and Functional Annotation- Dysregulation of genes involved in the Notch signaling system. **A** Conserved WD-40 domain (proteins involved in signal transduction, pre-mRNA processing and cytoskeleton assembly) **B** Regulators of ubiquitination of proteins and Membrane protein proteolysis involved in notch signaling.
(TIF)

S1 Table List of Primers and antibodies. (DOCX)

S2 Table > 2 log fold down regulated genes in microarray of DLD-1 cells under microgravity. (DOCX)

S3 Table > 2 log fold upregulated genes in microarray of DLD-1 cells under microgravity. (DOCX)

S4 Table > 2 log fold Up and down regulated genes in microarray of MOLT-4 cells under microgravity. (DOCX)

S5 Table Microarray analysis reveals commonly deregulated genes under Microgravity in both cell lines. (DOCX)
