# Supplementary figures and images for: Genome Wide Expression Profiling of Cancer Cell Lines Cultured in Microgravity Reveals Significant Dysregulation of Cell Cycle and MicroRNA Gene Networks
Source: PLoS One. 2015 Aug 21;10(8):e0135958. doi: 10.1371/journal.pone.0135958 (PMC4546578; doi:10.1371/journal.pone.0135958)

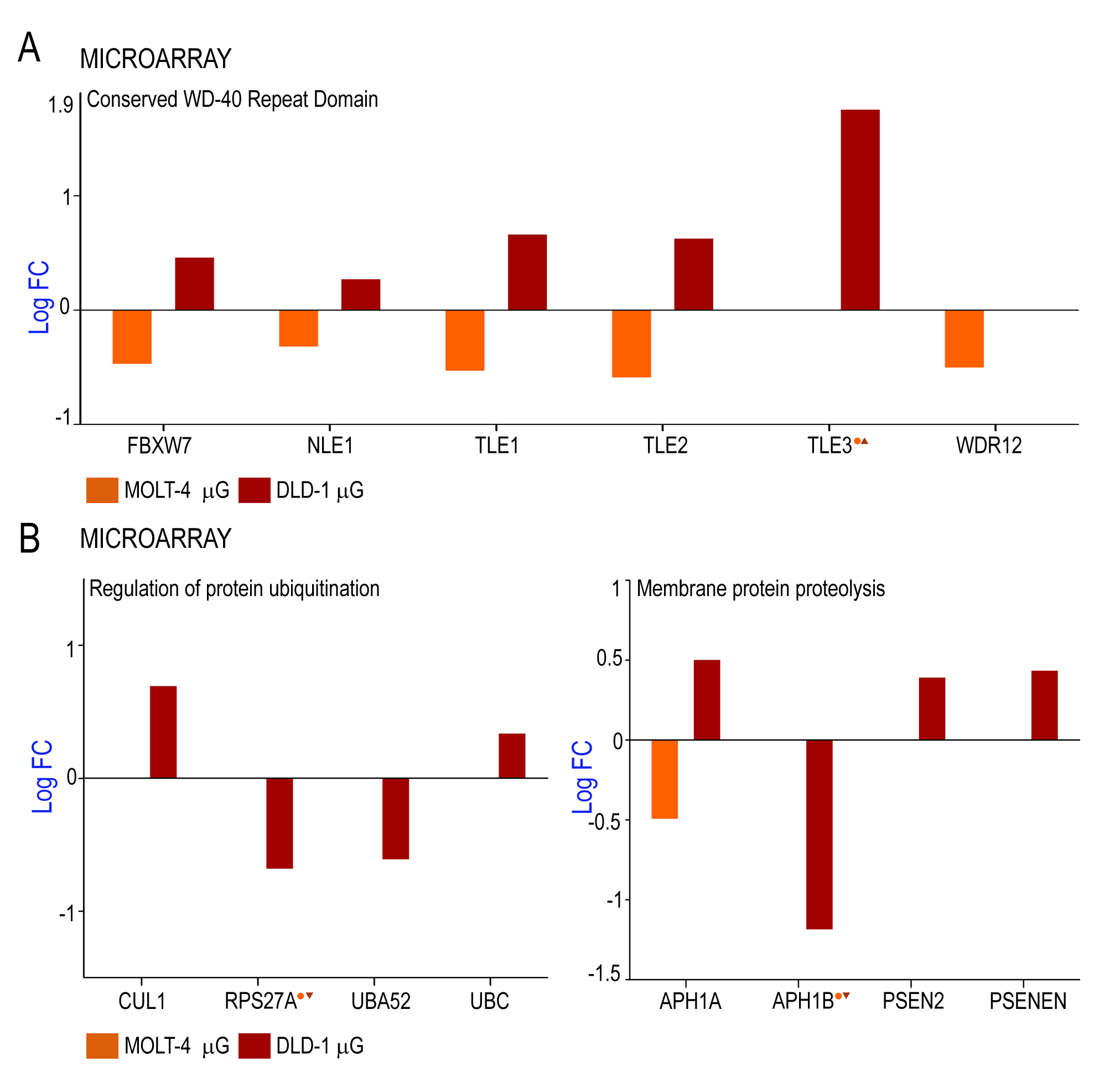

Supplement: S1 Fig — A Conserved WD-40 domain (proteins involved in signal transduction, pre-mRNA processing and cytoskeleton assembly) B Regulators of ubiquitination of proteins and Membrane protein proteolysis involved in notch signaling. (TIF) [file pone.0135958.s001.tif]
