# Supplementary material for: Genome Wide Expression Profiling of Cancer Cell Lines Cultured in Microgravity Reveals Significant Dysregulation of Cell Cycle and MicroRNA Gene Networks
Source: PLoS One. 2015 Aug 21;10(8):e0135958. doi: 10.1371/journal.pone.0135958 (PMC4546578; doi:10.1371/journal.pone.0135958)
Supplement: S1 Table — (DOCX) [file pone.0135958.s002.docx]

| **PRIMERS** |  |
| --- | --- |
| CCNB1 | FP 5’CGGGAAGTCACTGGAAACAT3’  RP 5’AAACATGGCAGTGACACCAA3’ |
| CCNE1 | FP 5’ATCCTCCAAAGTTGCACCAG3’  RP 5’AGGGGACTTAAACGCCACTT3’ |
| CDK1 | FP 5’TTTTCAGAGCTTTGGGCACT3’  RP 5’AGGCTTCCTGGTTTCCATTT3’ |
| CDK2 | FP 5’TTGTCAAGCTGCTGGATGTC3’  RP 5’TGATGAGGGGAAGAGGAATG3’ |
| MYC | FP 5’AGGCGAACACACAACGTCTT3’  RP 5’TTGGACGGACAGGATGTATGC3’ |
| ROMO1 | FP 5'CCGTCGTTTTCCGTGAGAGA3’  RP 5'ACCCATCACGAAGCCCATTT3’ |
| CD71 | FP 5'GCACCATCAAGCTGCTGAAT3’  RP 5'CGAGTTTTGAGCGCTGTCTT3’ |
| HES1 | FP 5’ AACACGACACCGGATAAACC3’  RP 5’TCAGCTGGCTCAGACTTTCA3’ |
| HEY1 | FP 5’GGAGTGTTGGTGGAAAGGAA3’  RP 5’CTCGCACACCATGATCACTT3’ |
| JUNB | FP 5’TGGTGGCCTCTCTCTACACGA3’  RP 5’GGGTCGGCCAGGTTGAC3’ |
| STAT3 | FP 5’GGCCCCTCGTCATCAAGA3’  RP 5’TTTGACCAGCAACCTGACTTTAGT3’ |
| CD105 | FP 5’CACTAGCCAGGTCTCGAAGG3’  RP 5’CTGAGGACCAGAAGCACCTC3’ |
| CD90 | FP 5' TCGCTCTCCTGCTAACAGTCT 3'  RP 5' CTCGTACTGGATGGGTGAACT 3' |
| CD117 | FP 5’AGCAAATCCATCCCCACACC3’  RP 5’GGCTTGAGCATCTTTACAGCGAC3’ |
| GAPDH | FP 5’ GAAGGTGAAGGTCGGAGT3’  RP 5’ GAAGATGGTGATGGGATTTC3’ |
| CCND1 | FP 5’ CCGTCCATGCGGAAGATC3’  RP 5’ ATGGCCAGCGGGAAGAC3’ |
| CDKN1A | FP 5’ TGGAGACTCTCAGGGTCGAAA3’  RP 5’ GGCGTTTGGAGTGGTAGAAATC3’ |
| **ANTIBODIES** |  |
| CD44 | Mol wt. 90-95 kDa, Dilution - 1:1000, Santa cruz, California USA Cat no. sc-71220 |
| Beta- Actin | Mol wt. 48 kDa, Dilution: 1:10000, Sigma Aldrich, USA Cat no. A5441 |
| **microRNA Primers** |  |
| U6 | FP 5’ CTGCGCAAGGATGACACGCA3’  FP 5’ GTGCAGGGTCCGAGGT3’ |
| miR-22 | FP 5’ AAGCTCCAGTTGAAGAACTGT3’  RP 5’AAGCTGCCAGTTGAAGAACTGT3’ |
