# Supplementary material for: Genome Wide Expression Profiling of Cancer Cell Lines Cultured in Microgravity Reveals Significant Dysregulation of Cell Cycle and MicroRNA Gene Networks
Source: PLoS One. 2015 Aug 21;10(8):e0135958. doi: 10.1371/journal.pone.0135958 (PMC4546578; doi:10.1371/journal.pone.0135958)
Supplement: S2 Table — (DOCX) [file pone.0135958.s003.docx]

| **> 2 log fold down regulated genes in microarray of DLD-1 cells under microgravity** | | | | | |
| --- | --- | --- | --- | --- | --- |
| **Fold Change**  **DLD-1**  **RCCS Vs Static** | **Log Fold Change**  **DLD-1**  **RCCS Vs Static** | **Reg.** | **Gene Symbol** | **Gene Title** | **Representative Public ID** |
| -18.0029 | **-4.17015** | down | **RRM2** | ribonucleotide reductase M2 | BQ050789 |
| -15.2369 | **-3.92949** | down | **METTL7A** | methyltransferase like 7A | AI073393 |
| -14.8356 | **-3.89099** | down | **TRIP13** | thyroid hormone receptor interactor 13 | U96131.1 |
| -14.3658 | **-3.84457** | down | **KIF14** | kinesin family member 14 | NM_014875.2 |
| -14.1801 | **-3.82579** | down | **CBX5** | chromobox homolog 5 | NM_001127322.1 |
| -13.6886 | **-3.7749** | down | **DTL** | denticleless E3 ubiquitin protein ligase homolog (Drosophila) | AI800576 |
| -13.6291 | **-3.76862** | down | **MCM3** | minichromosome maintenance complex component 3 | AK299566.1 |
| -13.0322 | **-3.70401** | down | **SPC24** | SPC24, NDC80 kinetochore complex component, homolog (S. cerevisiae) | AK075287.1 |
| -12.5053 | **-3.64447** | down | **ASPM** | asp (abnormal spindle) homolog, microcephaly associated (Drosophila) | AY971957.1 |
| -12.1251 | **-3.59993** | down | **DEPDC1** | DEP domain containing 1 | NM_017779.4 |
| -12.0682 | **-3.59314** | down | **CASC5** | cancer susceptibility candidate 5 | AB046790.1 |
| -11.748 | **-3.55435** | down | **MCM10** | minichromosome maintenance complex component 10 | NM_182751.1 |
| -11.3468 | **-3.50421** | down | **DIAPH3** | diaphanous homolog 3 (Drosophila) | NM_001042517.1 |
| -11.152 | **-3.47923** | down | **PRC1** | protein regulator of cytokinesis 1 | AK297117.1 |
| -11.0417 | **-3.46489** | down | **UHRF1** | ubiquitin-like with PHD and ring finger domains 1 | NM_013282.3 |
| -10.6581 | **-3.41388** | down | **FANCI** | Fanconi anemia, complementation group I | NM_018193.2 |
| -10.3621 | **-3.37325** | down | **RAD54L** | RAD54-like (S. cerevisiae) | AK292611.1 |
| -10.0062 | **-3.32282** | down | **CIT /// MIR1178** | citron (rho-interacting, serine/threonine kinase 21) /// microRNA 1178 | AY681966.1 |
| -9.70964 | **-3.27942** | down | **POLQ** | polymerase (DNA directed), theta | NM_199420.3 |
| -9.61976 | **-3.266** | down | **HELLS** | helicase, lymphoid-specific | AB102717.1 |
| -9.61066 | **-3.26464** | down | **MKI67** | antigen identified by monoclonal antibody Ki-67 | NM_002417.4 |
| -9.4398 | **-3.23876** | down | **KIAA1984** | KIAA1984 | BC007542.1 |
| -9.30513 | **-3.21803** | down | **TOP2A** | topoisomerase (DNA) II alpha 170kDa | J04088.1 |
| -9.17776 | **-3.19814** | down | **TYMS** | thymidylate synthetase | AB077208.1 |
| -9.15156 | **-3.19402** | down | **POLE2** | polymerase (DNA directed), epsilon 2, accessory subunit | AK293163.1 |
| -8.80343 | **-3.13807** | down | **FANCD2** | Fanconi anemia, complementation group D2 | AL832427.1 |
| -8.78977 | **-3.13583** | down | **RMI2** | RMI2, RecQ mediated genome instability 2, homolog (S. cerevisiae) | CR593562.1 |
| -8.78808 | **-3.13555** | down | **BRCA1** | breast cancer 1, early onset | NM_007295.2 |
| -8.57009 | **-3.09931** | down | **ARHGAP11A** | Rho GTPase activating protein 11A | NM_014783.3 |
| -8.39397 | **-3.06935** | down | **GINS2** | GINS complex subunit 2 (Psf2 homolog) | CR595781.1 |
| -8.08907 | **-3.01597** | down | **KIF20B** | kinesin family member 20B | NM_016195.2 |
| -7.97971 | **-2.99634** | down | **PFAS** | phosphoribosylformylglycinamidine synthase | NM_012393.2 |
| -7.95989 | **-2.99275** | down | **NCBP1** | nuclear cap binding protein subunit 1, 80kDa | DB308399 |
| -7.79509 | **-2.96257** | down | **C9orf100** | chromosome 9 open reading frame 100 | BC033666.1 |
| -7.56149 | **-2.91867** | down | **TTF2** | transcription termination factor, RNA polymerase II | NM_003594.3 |
| -7.44414 | **-2.8961** | down | **FANCA** | Fanconi anemia, complementation group A | CA309327 |
| -7.44142 | **-2.89558** | down | **CDC45** | cell division cycle 45 homolog (S. cerevisiae) | NM_003504.3 |
| -7.43727 | **-2.89477** | down | **KIF11** | kinesin family member 11 | NM_004523.3 |
| -7.4105 | **-2.88957** | down | **C16orf59** | chromosome 16 open reading frame 59 | BC008882.2 |
| -7.3723 | **-2.88212** | down | **CTPS1** | CTP synthase 1 | AL573032 |
| -7.34114 | **-2.876** | down | **BUB1B** | budding uninhibited by benzimidazoles 1 homolog beta (yeast) | NM_001211.5 |
| -7.27138 | **-2.86223** | down | **MCM4** | minichromosome maintenance complex component 4 | BG754783 |
| -7.26322 | **-2.86061** | down | **DHFR** | dihydrofolate reductase | NM_000791.3 |
| -7.18693 | **-2.84538** | down | **NCAPG2** | non-SMC condensin II complex, subunit G2 | NM_017760.5 |
| -7.13629 | **-2.83517** | down | **ZNF367** | zinc finger protein 367 | NM_153695.3 |
| -7.04775 | **-2.81716** | down | **CDC6** | cell division cycle 6 homolog (S. cerevisiae) | U77949.1 |
| -7.03492 | **-2.81453** | down | **ATAD2** | ATPase family, AAA domain containing 2 | NM_014109.3 |
| -7.02865 | **-2.81325** | down | **STIL** | SCL/TAL1 interrupting locus | BC144089.1 |
| -7.02774 | **-2.81306** | down | **RAD51** | RAD51 homolog (S. cerevisiae) | D13804.1 |
| -7.00585 | **-2.80856** | down | **RUVBL1** | RuvB-like 1 (E. coli) | DQ469310.1 |
| -7.00091 | **-2.80754** | down | **MYB** | v-myb myeloblastosis viral oncogene homolog (avian) | NM_001130173.1 |
| -6.99353 | **-2.80602** | down | **CENPA** | centromere protein A | BC002703.2 |
| -6.97707 | **-2.80262** | down | **MAML2** | mastermind-like 2 (Drosophila) | CR610618.1 |
| -6.916 | **-2.78994** | down | **FSBP /// RAD54B** | fibrinogen silencer binding protein /// RAD54 homolog B (S. cerevisiae) | NM_012415.2 |
| -6.80232 | **-2.76603** | down | **MIS18BP1** | MIS18 binding protein 1 | NM_018353.4 |
| -6.79903 | **-2.76533** | down | **TIAM1** | T-cell lymphoma invasion and metastasis 1 | NM_003253.2 |
| -6.79727 | **-2.76496** | down | **GINS1** | GINS complex subunit 1 (Psf1 homolog) | NM_021067.3 |
| -6.76439 | **-2.75796** | down | **POLA2** | polymerase (DNA directed), alpha 2, accessory subunit | AK297842.1 |
| -6.72355 | **-2.74922** | down | **CENPI** | centromere protein I | AK302986.1 |
| -6.66139 | **-2.73582** | down | **CDCA7** | cell division cycle associated 7 | DB364189 |
| -6.62396 | **-2.72769** | down | **CHEK1** | checkpoint kinase 1 | AI384092 |
| -6.61205 | **-2.7251** | down | **PHLDA1** | pleckstrin homology-like domain, family A, member 1 | BC110820.1 |
| -6.55992 | **-2.71368** | down | **CLSPN** | claspin | AY605064.1 |
| -6.525 | **-2.70598** | down | **DSN1** | DSN1, MIND kinetochore complex component, homolog (S. cerevisiae) | g223972617 |
| -6.4735 | **-2.69455** | down | **NCAPD3** | non-SMC condensin II complex, subunit D3 | NM_015261.2 |
| -6.46356 | **-2.69233** | down | **LGR5** | leucine-rich repeat containing G protein-coupled receptor 5 | CR600457.1 |
| -6.46126 | **-2.69181** | down | **ESPL1** | extra spindle pole bodies homolog 1 (S. cerevisiae) | NM_012291.4 |
| -6.45541 | **-2.69051** | down | **TRIM14** | tripartite motif containing 14 | NM_014788.2 |
| -6.41721 | **-2.68195** | down | **PARPBP** | PARP1 binding protein | BC098313.1 |
| -6.38205 | **-2.67402** | down | **PERP** | PERP, TP53 apoptosis effector | NM_022121.4 |
| -6.2541 | **-2.6448** | down | **LRP8** | low density lipoprotein receptor-related protein 8, apolipoprotein e receptor | NM_004631.3 |
| -6.19159 | **-2.63031** | down | **KIAA1524** | KIAA1524 | NM_020890.2 |
| -6.18191 | **-2.62805** | down | **KIAA1984** | KIAA1984 | BC007542.1 |
| -6.14827 | **-2.62018** | down | **KIF23** | kinesin family member 23 | AK308322.1 |
| -6.10175 | **-2.60922** | down | **SPC25** | SPC25, NDC80 kinetochore complex component, homolog (S. cerevisiae) | AF225416.1 |
| -6.06706 | **-2.601** | down | **DLGAP5** | discs, large (Drosophila) homolog-associated protein 5 | AK299338.1 |
| -6.0096 | **-2.58727** | down | **RCCD1** | RCC1 domain containing 1 | NM_033544.2 |
| -6.00536 | **-2.58625** | down | **NUSAP1** | nucleolar and spindle associated protein 1 | AK307728.1 |
| -5.98994 | **-2.58254** | down | **FANCB** | Fanconi anemia, complementation group B | NM_001018113.1 |
| -5.98781 | **-2.58203** | down | **CENPE** | centromere protein E, 312kDa | AB209996.1 |
| -5.97856 | **-2.5798** | down | **DDX11 /// DDX12P /// LOC642846** | DEAD/H (Asp-Glu-Ala-Asp/His) box helicase 11 /// DEAD/H (Asp-Glu-Ala-Asp/His) box polypeptide 12, pseudogene /// DEAD/H (Asp-Glu-Ala-Asp/His) box polypeptide 11-like | NM_030653.3 |
| -5.97371 | **-2.57863** | down | **WDHD1** | WD repeat and HMG-box DNA binding protein 1 | BC063041.1 |
| -5.91859 | **-2.56525** | down | **FAM173B** | family with sequence similarity 173, member B | AK316317.1 |
| -5.896 | **-2.55974** | down | **CKAP2** | cytoskeleton associated protein 2 | BC010901.1 |
| -5.89592 | **-2.55972** | down | **KIT** | v-kit Hardy-Zuckerman 4 feline sarcoma viral oncogene homolog | NM_000222.2 |
| -5.87115 | **-2.55364** | down | **OLFML3** | olfactomedin-like 3 | NM_020190.2 |
| -5.85887 | **-2.55062** | down | **WDR76** | WD repeat domain 76 | NM_024908.2 |
| -5.83535 | **-2.54482** | down | **ANK3** | ankyrin 3, node of Ranvier (ankyrin G) | NM_020987.2 |
| -5.82683 | **-2.54271** | down | **ABHD11** | abhydrolase domain containing 11 | AY053500.1 |
| -5.80958 | **-2.53843** | down | **DEPDC1B** | DEP domain containing 1B | AW181987 |
| -5.80794 | **-2.53803** | down | **EXO1** | exonuclease 1 | NM_130398.2 |
| -5.74239 | **-2.52165** | down | **SLC39A10** | solute carrier family 39 (zinc transporter), member 10 | NM_001127257.1 |
| -5.73177 | **-2.51898** | down | **C15orf42** | chromosome 15 open reading frame 42 | NM_152259.3 |
| -5.72674 | **-2.51771** | down | **KNTC1** | kinetochore associated 1 | NM_014708.4 |
| -5.71292 | **-2.51423** | down | **POLA1** | polymerase (DNA directed), alpha 1, catalytic subunit | NM_016937.2 |
| -5.63845 | **-2.4953** | down | **ATAD5** | ATPase family, AAA domain containing 5 | NM_024857.3 |
| -5.63188 | **-2.49362** | down | **PCP4** | Purkinje cell protein 4 | NM_006198.2 |
| -5.59745 | **-2.48477** | down | **MSH5 /// MSH5-SAPCD1 /// SAPCD1** | mutS homolog 5 (E. coli) /// MSH5-SAPCD1 readthrough (non-protein coding) /// suppressor APC domain containing 1 | CR596419.1 |
| -5.55987 | **-2.47505** | down | **USP13** | ubiquitin specific peptidase 13 (isopeptidase T-3) | NM_003940.2 |
| -5.55132 | **-2.47283** | down | **BUB1** | budding uninhibited by benzimidazoles 1 homolog (yeast) | AK302418.1 |
| -5.52391 | **-2.46569** | down | **ESCO2** | establishment of cohesion 1 homolog 2 (S. cerevisiae) | NM_001017420.2 |
| -5.52269 | **-2.46537** | down | **KIF2C** | kinesin family member 2C | AK290688.1 |
| -5.51332 | **-2.46292** | down | **ANLN** | anillin, actin binding protein | AF273437.1 |
| -5.49831 | **-2.45899** | down | **E2F8** | E2F transcription factor 8 | NM_024680.2 |
| -5.48853 | **-2.45642** | down | **PROM2** | prominin 2 | AA468507 |
| -5.48833 | **-2.45637** | down | **ASF1B** | ASF1 anti-silencing function 1 homolog B (S. cerevisiae) | AK302134.1 |
| -5.48795 | **-2.45627** | down | **DUT** | deoxyuridine triphosphatase | NM_001025248.1 |
| -5.44034 | **-2.4437** | down | **TMEM194A** | transmembrane protein 194A | NM_001130963.1 |
| -5.42878 | **-2.44063** | down | **TK1** | thymidine kinase 1, soluble | NM_003258.4 |
| -5.40113 | **-2.43326** | down | **HJURP** | Holliday junction recognition protein | AB162218.1 |
| -5.38682 | **-2.42943** | down | **C2orf18 /// CENPA** | chromosome 2 open reading frame 18 /// centromere protein A | NM_001042426.1 |
| -5.37384 | **-2.42595** | down | **CDCA2** | cell division cycle associated 2 | BC104451.1 |
| -5.36543 | **-2.42369** | down | **RAD51AP1** | RAD51 associated protein 1 | AF006259.1 |
| -5.3582 | **-2.42175** | down | **INF2** | inverted formin, FH2 and WH2 domain containing | NM_032714.1 |
| -5.34992 | **-2.41952** | down | **SGK494 /// SPAG5** | uncharacterized serine/threonine-protein kinase SgK494 /// sperm associated antigen 5 | AK316079.1 |
| -5.33519 | **-2.41554** | down | **SHCBP1** | SHC SH2-domain binding protein 1 | NM_024745.4 |
| -5.33194 | **-2.41466** | down | **C1orf112** | chromosome 1 open reading frame 112 | NM_018186.2 |
| -5.29521 | **-2.40469** | down | **PRTFDC1** | phosphoribosyl transferase domain containing 1 | AI983510 |
| -5.28318 | **-2.40141** | down | **TMTC4** | transmembrane and tetratricopeptide repeat containing 4 | NM_032813.2 |
| -5.2486 | **-2.39193** | down | **GINS4** | GINS complex subunit 4 (Sld5 homolog) | AK095334.1 |
| -5.23986 | **-2.38953** | down | **SMC2** | structural maintenance of chromosomes 2 | NM_006444.2 |
| -5.23131 | **-2.38717** | down | **C4orf21** | chromosome 4 open reading frame 21 | BX647594.1 |
| -5.214 | **-2.38239** | down | **MSMO1** | methylsterol monooxygenase 1 | BQ876670 |
| -5.2104 | **-2.38139** | down | **SORBS1** | sorbin and SH3 domain containing 1 | NM_001034954.1 |
| -5.20685 | **-2.38041** | down | **FAM64A** | family with sequence similarity 64, member A | NM_019013.1 |
| -5.19349 | **-2.3767** | down | **TRIM66** | tripartite motif containing 66 | NM_014818.1 |
| -5.18857 | **-2.37534** | down | **NEK2** | NIMA (never in mitosis gene a)-related kinase 2 | Z25425.1 |
| -5.16041 | **-2.36748** | down | **MELK** | maternal embryonic leucine zipper kinase | NM_014791.2 |
| -5.12577 | **-2.35777** | down | **ARL17A /// ARL17B /// LOC100294341** | ADP-ribosylation factor-like 17A /// ADP-ribosylation factor-like 17B /// ADP-ribosylation factor-like protein 17-like | BC041803.1 |
| -5.10382 | **-2.35158** | down | **OAS3** | 2'-5'-oligoadenylate synthetase 3, 100kDa | NM_006187.2 |
| -5.10298 | **-2.35134** | down | **ACAD9** | acyl-CoA dehydrogenase family, member 9 | AL524996 |
| -5.09368 | **-2.34871** | down | **ZWINT** | ZW10 interactor | CR624972.1 |
| -5.08114 | **-2.34515** | down | **CADPS** | Ca++-dependent secretion activator | NM_183393.2 |
| -5.06867 | **-2.34161** | down | **TMEM178A** | transmembrane protein 178A | AI609358 |
| -5.06214 | **-2.33975** | down | **PIF1** | PIF1 5'-to-3' DNA helicase homolog (S. cerevisiae) | NM_025049.2 |
| -5.05718 | **-2.33833** | down | **MIR21** | microRNA 21 | AA837010 |
| -5.02722 | **-2.32976** | down | **C18orf54** | chromosome 18 open reading frame 54 | BF059556 |
| -4.97958 | **-2.31602** | down | **TCF19** | transcription factor 19 | BC044632.1 |
| -4.97293 | **-2.3141** | down | **WHSC1** | Wolf-Hirschhorn syndrome candidate 1 | CA430145 |
| -4.96116 | **-2.31068** | down | **ANKRD22** | ankyrin repeat domain 22 | BC021671.1 |
| -4.90457 | **-2.29413** | down | **E2F7** | E2F transcription factor 7 | NM_203394.2 |
| -4.90085 | **-2.29303** | down | **SPATA5** | spermatogenesis associated 5 | BU618411 |
| -4.89316 | **-2.29077** | down | **PRSS23** | protease, serine, 23 | NM_007173.4 |
| -4.89038 | **-2.28995** | down | **BIRC5** | baculoviral IAP repeat containing 5 | AK301793.1 |
| -4.8877 | **-2.28916** | down | **NSMCE4A** | non-SMC element 4 homolog A (S. cerevisiae) | CX759064 |
| -4.86595 | **-2.28272** | down | **DIDO1** | death inducer-obliterator 1 | BC000770.2 |
| -4.83192 | **-2.2726** | down | **STXBP6** | syntaxin binding protein 6 (amisyn) | AL834346.1 |
| -4.81353 | **-2.2671** | down | **EMG1** | EMG1 nucleolar protein homolog (S. cerevisiae) | NM_006331.6 |
| -4.7851 | **-2.25855** | down | **C4orf46** | chromosome 4 open reading frame 46 | NM_001008393.2 |
| -4.7694 | **-2.25381** | down | **RFC5** | replication factor C (activator 1) 5, 36.5kDa | L07540.1 |
| -4.76124 | **-2.25134** | down | **UBASH3B** | ubiquitin associated and SH3 domain containing B | NM_032873.4 |
| -4.75683 | **-2.25** | down | **ODZ3** | odz, odd Oz/ten-m homolog 3 (Drosophila) | BU623004 |
| -4.75543 | **-2.24957** | down | **TIMELESS** | timeless homolog (Drosophila) | BC050557.1 |
| -4.75368 | **-2.24904** | down | **SGOL2** | shugoshin-like 2 (S. pombe) | BC092412.1 |
| -4.73031 | **-2.24193** | down | **BLM** | Bloom syndrome, RecQ helicase-like | BC143288.1 |
| -4.72583 | **-2.24057** | down | **SLC4A7** | solute carrier family 4, sodium bicarbonate cotransporter, member 7 | NM_003615.3 |
| -4.72288 | **-2.23967** | down | **AURKB** | aurora kinase B | NM_004217.2 |
| -4.72109 | **-2.23912** | down | **NSUN5P1 /// NSUN5P2** | NOP2/Sun domain family, member 5 pseudogene 1 /// NOP2/Sun domain family, member 5 pseudogene 2 | BC101515.1 |
| -4.71989 | **-2.23875** | down | **PRKDC** | protein kinase, DNA-activated, catalytic polypeptide | U34994.3 |
| -4.70675 | **-2.23473** | down | **RAB26** | RAB26, member RAS oncogene family | NM_014353.4 |
| -4.70399 | **-2.23388** | down | **PHGR1** | proline/histidine/glycine-rich 1 | NM_001145643.1 |
| -4.68985 | **-2.22954** | down | **DNMT1** | DNA (cytosine-5-)-methyltransferase 1 | BF037423 |
| -4.68867 | **-2.22918** | down | **KIAA0101** | KIAA0101 | NM_014736.4 |
| -4.68292 | **-2.22741** | down | **POLR3K** | polymerase (RNA) III (DNA directed) polypeptide K, 12.3 kDa | BU623690 |
| -4.67003 | **-2.22343** | down | **MCM7** | minichromosome maintenance complex component 7 | AF279900.1 |
| -4.65679 | **-2.21934** | down | **SPAG5** | sperm associated antigen 5 | NM_006461.3 |
| -4.65485 | **-2.21874** | down | **UGT8** | UDP glycosyltransferase 8 | AL118812 |
| -4.63738 | **-2.21331** | down | **GSPT1** | G1 to S phase transition 1 | AW972533 |
| -4.63033 | **-2.21111** | down | **RAD1** | RAD1 homolog (S. pombe) | NM_002853.3 |
| -4.62838 | **-2.21051** | down | **C11orf82** | chromosome 11 open reading frame 82 | NM_145018.3 |
| -4.61019 | **-2.20483** | down | **MTPAP** | mitochondrial poly(A) polymerase | NM_018109.3 |
| -4.60178 | **-2.20219** | down | **HPSE** | heparanase | AF155510.1 |
| -4.59796 | **-2.201** | down | **CDK1** | cyclin-dependent kinase 1 | NM_001786.3 |
| -4.59561 | **-2.20026** | down | **TMPO** | thymopoietin | AB209297.1 |
| -4.58205 | **-2.19599** | down | **CORO1A** | coronin, actin binding protein, 1A | BC126387.1 |
| -4.57727 | **-2.19449** | down | **DEPDC4** | DEP domain containing 4 | BC100928.2 |
| -4.54501 | **-2.18428** | down | **CENPM** | centromere protein M | NM_001110215.1 |
| -4.54236 | **-2.18344** | down | **CSTF3** | cleavage stimulation factor, 3' pre-RNA, subunit 3, 77kDa | NM_001033505.1 |
| -4.53921 | **-2.18244** | down | **BDH1** | 3-hydroxybutyrate dehydrogenase, type 1 | BC019317.1 |
| -4.53323 | **-2.18054** | down | **CHTF18** | CTF18, chromosome transmission fidelity factor 18 homolog (S. cerevisiae) | BC006437.1 |
| -4.50893 | **-2.17279** | down | **KIFC1** | kinesin family member C1 | NM_002263.3 |
| -4.50117 | **-2.1703** | down | **MTHFD1** | methylenetetrahydrofolate dehydrogenase (NADP+ dependent) 1, methenyltetrahydrofolate cyclohydrolase, formyltetrahydrofolate synthetase | NM_005956.3 |
| -4.49368 | **-2.1679** | down | **CEP72** | centrosomal protein 72kDa | BQ771509 |
| -4.46907 | **-2.15998** | down | **SLC7A2** | solute carrier family 7 (cationic amino acid transporter, y+ system), member 2 | CA449960 |
| -4.46761 | **-2.1595** | down | **MSH2** | mutS homolog 2, colon cancer, nonpolyposis type 1 (E. coli) | AK299667.1 |
| -4.46123 | **-2.15744** | down | **KDELC2** | KDEL (Lys-Asp-Glu-Leu) containing 2 | NM_153705.4 |
| -4.43863 | **-2.15011** | down | **CKAP2L** | cytoskeleton associated protein 2-like | NM_152515.3 |
| -4.432 | **-2.14796** | down | **TRIOBP** | TRIO and F-actin binding protein | NM_138632.2 |
| -4.42746 | **-2.14648** | down | **GNE** | glucosamine (UDP-N-acetyl)-2-epimerase/N-acetylmannosamine kinase | NM_005476.4 |
| -4.38868 | **-2.13379** | down | **ERCC6L** | excision repair cross-complementing rodent repair deficiency, complementation group 6-like | NM_017669.2 |
| -4.38357 | **-2.13211** | down | **CD24** | CD24 molecule | NM_013230.2 |
| -4.36486 | **-2.12593** | down | **FAM72A /// FAM72B /// FAM72D** | family with sequence similarity 72, member A /// family with sequence similarity 72, member B /// family with sequence similarity 72, member D | BC137546.1 |
| -4.36387 | **-2.12561** | down | **C2orf43** | chromosome 2 open reading frame 43 | NM_021925.2 |
| -4.35666 | **-2.12322** | down | **TRIM34 /// TRIM6-TRIM34** | tripartite motif containing 34 /// TRIM6-TRIM34 readthrough | NM_001003819.2 |
| -4.33151 | **-2.11487** | down | **CDC7** | cell division cycle 7 homolog (S. cerevisiae) | NM_003503.3 |
| -4.31137 | **-2.10815** | down | **CCNA2** | cyclin A2 | NM_001237.3 |
| -4.30961 | **-2.10756** | down | **RNASEH2A** | ribonuclease H2, subunit A | CR597970.1 |
| -4.30135 | **-2.10479** | down | **RFX5** | regulatory factor X, 5 (influences HLA class II expression) | BQ686723 |
| -4.29921 | **-2.10407** | down | **WHSC1** | Wolf-Hirschhorn syndrome candidate 1 | BC141815.1 |
| -4.28763 | **-2.10018** | down | **SRSF11** | serine/arginine-rich splicing factor 11 | AU146237 |
| -4.28001 | **-2.09761** | down | **CENPO** | centromere protein O | NM_024322.1 |
| -4.24996 | **-2.08745** | down | **HOXA13** | homeobox A13 | BC075791.1 |
| -4.19094 | **-2.06728** | down | **CEP55** | centrosomal protein 55kDa | NM_018131.4 |
| -4.1895 | **-2.06678** | down | **CHRNA1** | cholinergic receptor, nicotinic, alpha 1 (muscle) | AK299445.1 |
| -4.18072 | **-2.06375** | down | **MECR** | mitochondrial trans-2-enoyl-CoA reductase | AK300889.1 |
| -4.15798 | **-2.05588** | down | **ADRB1** | adrenoceptor beta 1 | NM_000684.2 |
| -4.15789 | **-2.05585** | down | **TMEM48** | transmembrane protein 48 | AL354613.1 |
| -4.15009 | **-2.05314** | down | **HOOK3** | hook homolog 3 (Drosophila) | BF447112 |
| -4.14067 | **-2.04986** | down | **CXCL5** | chemokine (C-X-C motif) ligand 5 | CD367695 |
| -4.1321 | **-2.04687** | down | **LEPR** | leptin receptor | NM_001003679.1 |
| -4.1227 | **-2.04359** | down | **CCNF** | cyclin F | NM_001761.2 |
| -4.12203 | **-2.04335** | down | **HPGD** | hydroxyprostaglandin dehydrogenase 15-(NAD) | DB377018 |
| -4.11978 | **-2.04257** | down | **STMN1** | stathmin 1 | AB451319.1 |
| -4.11935 | **-2.04242** | down | **HSPA8 /// SNORD14C /// SNORD14D** | heat shock 70kDa protein 8 /// small nucleolar RNA, C/D box 14C /// small nucleolar RNA, C/D box 14D | NR_001454.1 |
| -4.11405 | **-2.04056** | down | **SRSF1** | serine/arginine-rich splicing factor 1 | AI364887 |
| -4.11152 | **-2.03967** | down | **HNRNPD** | heterogeneous nuclear ribonucleoprotein D (AU-rich element RNA binding protein 1, 37kDa) | AK303552.1 |
| -4.09524 | **-2.03395** | down | **WDR90** | WD repeat domain 90 | NM_145294.4 |
| -4.09226 | **-2.0329** | down | **KLF12** | Kruppel-like factor 12 | AF330041.1 |
| -4.09218 | **-2.03287** | down | **MCM2** | minichromosome maintenance complex component 2 | AK304204.1 |
| -4.0839 | **-2.02995** | down | **CAMK2D** | calcium/calmodulin-dependent protein kinase II delta | NM_001221.3 |
| -4.06451 | **-2.02308** | down | **KATNAL1** | katanin p60 subunit A-like 1 | NM_032116.3 |
| -4.0565 | **-2.02024** | down | **MCCC2** | methylcrotonoyl-CoA carboxylase 2 (beta) | NM_022132.4 |
| -4.04728 | **-2.01695** | down | **GRSF1** | G-rich RNA sequence binding factor 1 | NM_002092.3 |
| -4.04184 | **-2.01501** | down | **C18orf54** | chromosome 18 open reading frame 54 | NM_173529.4 |
| -4.03689 | **-2.01324** | down | **NCAPH** | non-SMC condensin I complex, subunit H | BC024211.2 |
| -4.03683 | **-2.01322** | down | **SKA3** | spindle and kinetochore associated complex subunit 3 | BC048988.1 |
| -4.03514 | **-2.01262** | down | **ARHGAP11A /// ARHGAP11B /// LOC100288637** | Rho GTPase activating protein 11A /// Rho GTPase activating protein 11B /// OTU domain containing 7A pseudogene | NM_001039841.1 |
| -4.03005 | **-2.0108** | down | **AGPS** | alkylglycerone phosphate synthase | NM_003659.3 |
| -4.02619 | **-2.00941** | down | **HAUS5** | HAUS augmin-like complex, subunit 5 | NM_015302.1 |
| -4.01877 | **-2.00675** | down | **CLDN2** | claudin 2 | NM_020384.2 |
| -4.002 | **-2.00072** | down | **PPP1R9A** | protein phosphatase 1, regulatory subunit 9A | NM_017650.2 |
| -4.00115 | **-2.00041** | down | **KIF4A /// KIF4B** | kinesin family member 4A /// kinesin family member 4B | NM_001099293.1 |
