# Supplementary material for: Genome Wide Expression Profiling of Cancer Cell Lines Cultured in Microgravity Reveals Significant Dysregulation of Cell Cycle and MicroRNA Gene Networks
Source: PLoS One. 2015 Aug 21;10(8):e0135958. doi: 10.1371/journal.pone.0135958 (PMC4546578; doi:10.1371/journal.pone.0135958)
Supplement: S3 Table — (DOCX) [file pone.0135958.s004.docx]

| **> 2 log fold upregulated genes in microarray of DLD-1 cells under microgravity** | | | | | |
| --- | --- | --- | --- | --- | --- |
| **Fold Change**  **DLD-1**  **RCCS Vs Static** | **Log Fold Change**  **DLD-1**  **RCCS Vs Static** | **Reg.** | **Gene Symbol** | **Gene Title** | **Representative Public ID** |
| 97.24456 | **6.6035457** | up | **SPINK1** | serine peptidase inhibitor, Kazal type 1 | NM_003122.3 |
| 21.302397 | **4.412944** | up | **MIR22 /// MIR22HG** | microRNA 22 /// MIR22 host gene (non-protein coding) | CA305977 |
| 15.889903 | **3.9900384** | up | **GNG2** | guanine nucleotide binding protein (G protein), gamma 2 | BU146364 |
| 13.189001 | **3.7212634** | up | **CPEB2** | cytoplasmic polyadenylation element binding protein 2 | AY247744.1 |
| 12.984857 | **3.6987581** | up | **UPP1** | uridine phosphorylase 1 | AK297863.1 |
| 12.368876 | **3.6286426** | up | **ARRDC3** | arrestin domain containing 3 | NM_020801.2 |
| 11.989501 | **3.5836997** | up | **HIST1H2BD** | histone cluster 1, H2bd | NM_138720.1 |
| 10.94926 | **3.4527614** | up | **PRSS1** | protease, serine, 1 (trypsin 1) | NM_002769.3 |
| 10.795611 | **3.432373** | up | **MOB1B** | MOB kinase activator 1B | AK299481.1 |
| 10.385803 | **3.376541** | up | **KLHL11** | kelch-like 11 (Drosophila) | NM_018143.1 |
| 9.956728 | **3.3156717** | up | **HIST1H4A /// HIST1H4B /// HIST1H4C /// HIST1H4D /// HIST1H4E /// HIST1H4F /// HIST1H4H /// HIST1H4I /// HIST1H4J /// HIST1H4K /// HIST1H4L /// HIST2H4A /// HIST2H4B /// HIST4H4** | histone cluster 1, H4a /// histone cluster 1, H4b /// histone cluster 1, H4c /// histone cluster 1, H4d /// histone cluster 1, H4e /// histone cluster 1, H4f /// histone cluster 1, H4h /// histone cluster 1, H4i /// histone cluster 1, H4j /// histone cluster 1, H4k /// histone cluster 1, H4l /// histone cluster 2, H4a /// histone cluster 2, H4b /// histone cluster 4, H4 | NM_003543.3 |
| 9.936786 | **3.3127792** | up | **BHLHE41** | basic helix-loop-helix family, member e41 | AI242138 |
| 9.839668 | **3.2986097** | up | **NR4A3** | nuclear receptor subfamily 4, group A, member 3 | NM_173198.1 |
| 9.653494 | **3.2710512** | up | **HMOX1** | heme oxygenase (decycling) 1 | NM_002133.1 |
| 9.567625 | **3.2581608** | up | **EIF4E** | eukaryotic translation initiation factor 4E | BG723974 |
| 9.31595 | **3.219703** | up | **GADD45G** | growth arrest and DNA-damage-inducible, gamma | CR613579.1 |
| 8.881 | **3.150722** | up | **HES1** | hairy and enhancer of split 1, (Drosophila) | CD236691 |
| 8.86245 | **3.1477056** | up | **HIST1H3A /// HIST1H3B /// HIST1H3C /// HIST1H3D /// HIST1H3E /// HIST1H3F /// HIST1H3G /// HIST1H3H /// HIST1H3I /// HIST1H3J** | histone cluster 1, H3a /// histone cluster 1, H3b /// histone cluster 1, H3c /// histone cluster 1, H3d /// histone cluster 1, H3e /// histone cluster 1, H3f /// histone cluster 1, H3g /// histone cluster 1, H3h /// histone cluster 1, H3i /// histone cluster 1, H3j | NM_003537.3 |
| 8.583593 | **3.1015818** | up | **DNAJC24** | DnaJ (Hsp40) homolog, subfamily C, member 24 | AK289665.1 |
| 8.58126 | **3.1011894** | up | **DNAJC3** | DnaJ (Hsp40) homolog, subfamily C, member 3 | BC047936.1 |
| 8.534584 | **3.0933208** | up | **OSTBETA** | organic solute transporter beta | g31341260 |
| 8.494914 | **3.0865993** | up | **PRSS1 /// PRSS2 /// PRSS3P2** | protease, serine, 1 (trypsin 1) /// protease, serine, 2 (trypsin 2) /// protease, serine, 3 pseudogene 2 | BP325533 |
| 8.37404 | **3.0659237** | up | **HBEGF** | heparin-binding EGF-like growth factor | NM_001945.2 |
| 8.182056 | **3.0324636** | up | **C3orf58** | chromosome 3 open reading frame 58 | AK294020.1 |
| 8.101304 | **3.0181541** | up | **HIST1H2BC /// HIST1H2BE /// HIST1H2BF /// HIST1H2BG /// HIST1H2BI** | histone cluster 1, H2bc /// histone cluster 1, H2be /// histone cluster 1, H2bf /// histone cluster 1, H2bg /// histone cluster 1, H2bi | NM_003526.2 |
| 8.100454 | **3.0180027** | up | **CLEC2B** | C-type lectin domain family 2, member B | BC005254.1 |
| 8.049454 | **3.0088909** | up | **RGS16** | regulator of G-protein signaling 16 | AK301261.1 |
| 7.9264507 | **2.986675** | up | **BROX** | BRO1 domain and CAAX motif containing | NM_144695.2 |
| 7.798181 | **2.9631376** | up | **EGLN1** | egl nine homolog 1 (C. elegans) | BC005369.1 |
| 7.5582848 | **2.9180589** | up | **PRSS2 /// PRSS3** | protease, serine, 2 (trypsin 2) /// protease, serine, 3 | BP325533 |
| 7.4401937 | **2.8953402** | up | **CCDC75** | coiled-coil domain containing 75 | AK289504.1 |
| 7.3415117 | **2.8760772** | up | **FAM83B** | family with sequence similarity 83, member B | NM_001010872.1 |
| 7.189769 | **2.8459454** | up | **JUN** | jun proto-oncogene | BG491844 |
| 7.147459 | **2.8374305** | up | **TRPS1** | trichorhinophalangeal syndrome I | AK000948.1 |
| 7.113824 | **2.8306253** | up | **NBR2** | neighbor of BRCA1 gene 2 (non-protein coding) | AY597810.1 |
| 6.956979 | **2.798461** | up | **BHLHE40** | basic helix-loop-helix family, member e40 | BC082238.1 |
| 6.9326525 | **2.7934074** | up | **DIDO1** | death inducer-obliterator 1 | BX097024 |
| 6.8693624 | **2.7801762** | up | **LATS1** | LATS, large tumor suppressor, homolog 1 (Drosophila) | AF104413.1 |
| 6.836364 | **2.7732291** | up | **SIAH2** | siah E3 ubiquitin protein ligase 2 | BX373747 |
| 6.823917 | **2.7706** | up | **NOV** | nephroblastoma overexpressed | AY082381.1 |
| 6.7533937 | **2.7556126** | up | **SPRR2D** | small proline-rich protein 2D | NM_006945.4 |
| 6.6120753 | **2.7251031** | up | **CBFB** | core-binding factor, beta subunit | AK291834.1 |
| 6.525414 | **2.7060695** | up | **MYCL1** | v-myc myelocytomatosis viral oncogene homolog 1, lung carcinoma derived (avian) | AK296078.1 |
| 6.4580984 | **2.6911094** | up | **FOSL2** | FOS-like antigen 2 | AI860150 |
| 6.4409904 | **2.6872826** | up | **ATF3** | activating transcription factor 3 | NM_001040619.1 |
| 6.43964 | **2.68698** | up | **STK32A** | serine/threonine kinase 32A | NM_145001.3 |
| 6.311106 | **2.657893** | up | **FBXO32** | F-box protein 32 | NM_058229.2 |
| 6.248747 | **2.6435668** | up | **KHDRBS3** | KH domain containing, RNA binding, signal transduction associated 3 | BC032606.2 |
| 6.224735 | **2.6380124** | up | **IL1R1** | interleukin 1 receptor, type I | NM_000877.2 |
| 6.1375237 | **2.6176567** | up | **EIF5** | eukaryotic translation initiation factor 5 | AI033691 |
| 6.094872 | **2.607596** | up | **IGFBP3** | insulin-like growth factor binding protein 3 | BC064987.1 |
| 6.0686326 | **2.6013715** | up | **MIR22 /// MIR22HG** | microRNA 22 /// MIR22 host gene (non-protein coding) | BC119720.1 |
| 6.0550675 | **2.598143** | up | **DLX2** | distal-less homeobox 2 | NM_004405.3 |
| 6.0305147 | **2.592281** | up | **GPRC5C** | G protein-coupled receptor, family C, group 5, member C | NM_022036.2 |
| 5.9989576 | **2.5847118** | up | **SH3BGRL2** | SH3 domain binding glutamic acid-rich protein like 2 | DB355115 |
| 5.9280334 | **2.5675535** | up | **IRS2** | insulin receptor substrate 2 | BM801836 |
| 5.9154835 | **2.564496** | up | **CDC14B** | CDC14 cell division cycle 14 homolog B (S. cerevisiae) | EF611343.1 |
| 5.912015 | **2.56365** | up | **C21orf91** | chromosome 21 open reading frame 91 | CR457317.1 |
| 5.885304 | **2.557117** | up | **SLC9A2** | solute carrier family 9, subfamily A (NHE2, cation proton antiporter 2), member 2 | NM_003048.3 |
| 5.877142 | **2.5551147** | up | **CEBPG** | CCAAT/enhancer binding protein (C/EBP), gamma | U20240.1 |
| 5.847056 | **2.5477104** | up | **SLC3A2** | solute carrier family 3 (activators of dibasic and neutral amino acid transport), member 2 | J03569.1 |
| 5.7828484 | **2.5317802** | up | **FEM1B** | fem-1 homolog b (C. elegans) | NM_015322.3 |
| 5.771823 | **2.529027** | up | **ITPR1** | inositol 1,4,5-trisphosphate receptor, type 1 | BM992183 |
| 5.7560773 | **2.525086** | up | **CHST3** | carbohydrate (chondroitin 6) sulfotransferase 3 | NM_004273.4 |
| 5.7339635 | **2.5195327** | up | **WRAP53** | WD repeat containing, antisense to TP53 | U58658.1 |
| 5.716149 | **2.5150435** | up | **PLEKHF2** | pleckstrin homology domain containing, family F (with FYVE domain) member 2 | CR615432.1 |
| 5.671125 | **2.503635** | up | **C6orf52** | chromosome 6 open reading frame 52 | AW207788 |
| 5.6477532 | **2.497677** | up | **MIER1** | mesoderm induction early response 1 homolog (Xenopus laevis) | AK302061.1 |
| 5.617346 | **2.4898887** | up | **CCDC126** | coiled-coil domain containing 126 | BF062262 |
| 5.57087 | **2.4779027** | up | **ATP6V0D2** | ATPase, H+ transporting, lysosomal 38kDa, V0 subunit d2 | AY079172.1 |
| 5.565889 | **2.476612** | up | **ZNF425** | zinc finger protein 425 | NM_001001661.1 |
| 5.5646143 | **2.4762816** | up | **KIF27** | kinesin family member 27 | AY237537.1 |
| 5.53181 | **2.4677515** | up | **TMEM167B** | transmembrane protein 167B | BC146873.1 |
| 5.527935 | **2.4667406** | up | **INSL4** | insulin-like 4 (placenta) | NM_002195.1 |
| 5.523852 | **2.4656746** | up | **DLL1** | delta-like 1 (Drosophila) | NM_005618.3 |
| 5.4785895 | **2.4538045** | up | **CCRN4L** | CCR4 carbon catabolite repression 4-like (S. cerevisiae) | BC023512.2 |
| 5.4775963 | **2.453543** | up | **HOXC6** | homeobox C6 | M16938.1 |
| 5.475371 | **2.4529567** | up | **TMEM170A** | transmembrane protein 170A | NM_145254.1 |
| 5.4397435 | **2.4435387** | up | **ZSCAN29** | zinc finger and SCAN domain containing 29 | AF525399.1 |
| 5.4372845 | **2.4428864** | up | **ZBTB6** | zinc finger and BTB domain containing 6 | AK292401.1 |
| 5.411157 | **2.4359372** | up | **MAF** | v-maf musculoaponeurotic fibrosarcoma oncogene homolog (avian) | BI492527 |
| 5.3903265 | **2.4303727** | up | **HSPA6** | heat shock 70kDa protein 6 (HSP70B') | BC035665.1 |
| 5.3739786 | **2.4259906** | up | **C14orf182** | chromosome 14 open reading frame 182 | BC128083.1 |
| 5.338733 | **2.4164975** | up | **TADA2B** | transcriptional adaptor 2B | BC101334.1 |
| 5.293915 | **2.404335** | up | **MGARP** | mitochondria-localized glutamic acid-rich protein | NM_032623.3 |
| 5.282341 | **2.4011774** | up | **MFAP3** | microfibrillar-associated protein 3 | BC026244.1 |
| 5.2681646 | **2.3973005** | up | **HIST1H2AD /// HIST1H3A /// HIST1H3B /// HIST1H3C /// HIST1H3D /// HIST1H3E /// HIST1H3F /// HIST1H3G /// HIST1H3H /// HIST1H3I /// HIST1H3J** | histone cluster 1, H2ad /// histone cluster 1, H3a /// histone cluster 1, H3b /// histone cluster 1, H3c /// histone cluster 1, H3d /// histone cluster 1, H3e /// histone cluster 1, H3f /// histone cluster 1, H3g /// histone cluster 1, H3h /// histone cluster 1, H3i /// histone cluster 1, H3j | BC093809.1 |
| 5.245415 | **2.391057** | up | **AQP11** | aquaporin 11 | NM_173039.1 |
| 5.238473 | **2.3891463** | up | **HIPK3** | homeodomain interacting protein kinase 3 | AK304063.1 |
| 5.224055 | **2.38517** | up | **NRIP1** | nuclear receptor interacting protein 1 | BC040361.1 |
| 5.2238984 | **2.3851268** | up | **CHD2** | chromodomain helicase DNA binding protein 2 | NM_001042572.2 |
| 5.2094584 | **2.3811333** | up | **HIST1H2AD** | histone cluster 1, H2ad | BC093809.1 |
| 5.1872673 | **2.3749747** | up | **DNAJB9** | DnaJ (Hsp40) homolog, subfamily B, member 9 | BX411686 |
| 5.1844068 | **2.374179** | up | **HIST1H1C** | histone cluster 1, H1c | NM_005319.3 |
| 5.1501365 | **2.3646107** | up | **SLC7A11** | solute carrier family 7 (anionic amino acid transporter light chain, xc- system), member 11 | AB040875.1 |
| 5.1334157 | **2.359919** | up | **FAM168B** | family with sequence similarity 168, member B | BF056651 |
| 5.104226 | **2.3516922** | up | **HSPA4L** | heat shock 70kDa protein 4-like | NM_014278.2 |
| 5.100978 | **2.3507738** | up | **TRAM1** | translocation associated membrane protein 1 | AK292777.1 |
| 5.073168 | **2.342887** | up | **ABHD5** | abhydrolase domain containing 5 | BC021958.1 |
| 5.0358734 | **2.332242** | up | **CCSAP** | centriole, cilia and spindle-associated protein | AK291437.1 |
| 5.0358486 | **2.3322349** | up | **PPP3R1** | protein phosphatase 3, regulatory subunit B, alpha | CB243698 |
| 4.9886518 | **2.31865** | up | **WASL** | Wiskott-Aldrich syndrome-like | D88460.1 |
| 4.9784765 | **2.3157043** | up | **TAF13** | TAF13 RNA polymerase II, TATA box binding protein (TBP)-associated factor, 18kDa | BC121180.1 |
| 4.971615 | **2.3137145** | up | **THAP2** | THAP domain containing, apoptosis associated protein 2 | CR533485.1 |
| 4.968431 | **2.3127904** | up | **UBE2J1** | ubiquitin-conjugating enzyme E2, J1 | BC013973.1 |
| 4.963681 | **2.3114104** | up | **HBS1L** | HBS1-like (S. cerevisiae) | NM_001145207.1 |
| 4.93369 | **2.3026671** | up | **SOCS1** | suppressor of cytokine signaling 1 | NM_003745.1 |
| 4.9091473 | **2.2954724** | up | **SERPINB8** | serpin peptidase inhibitor, clade B (ovalbumin), member 8 | AK300391.1 |
| 4.907725 | **2.2950544** | up | **MMP1** | matrix metallopeptidase 1 (interstitial collagenase) | AK098450.1 |
| 4.8733354 | **2.2849095** | up | **ENPP4** | ectonucleotide pyrophosphatase/phosphodiesterase 4 (putative) | BC018054.1 |
| 4.867763 | **2.283259** | up | **MIR17HG /// MIR18A /// MIR19A /// MIR19B1 /// MIR20A /// MIR92A1** | miR-17-92 cluster host gene (non-protein coding) /// microRNA 18a /// microRNA 19a /// microRNA 19b-1 /// microRNA 20a /// microRNA 92a-1 | AB176707.1 |
| 4.8498425 | **2.277938** | up | **ULBP1** | UL16 binding protein 1 | NM_025218.2 |
| 4.825789 | **2.2707648** | up | **RCL1** | RNA terminal phosphate cyclase-like 1 | AL582781 |
| 4.8240786 | **2.2702534** | up | **PER2** | period homolog 2 (Drosophila) | BM726973 |
| 4.8201666 | **2.269083** | up | **HIST1H2BK** | histone cluster 1, H2bk | NM_080593.1 |
| 4.813144 | **2.2669797** | up | **FGF7 /// KGFLP1 /// KGFLP2** | fibroblast growth factor 7 /// fibroblast growth factor 7 pseudogene /// keratinocyte growth factor-like protein 2 | AF523265.1 |
| 4.8027906 | **2.2638729** | up | **SNX18** | sorting nexin 18 | BC060791.1 |
| 4.7782636 | **2.2564864** | up | **ZNF503** | zinc finger protein 503 | BC011625.2 |
| 4.7754035 | **2.2556226** | up | **BCL2L11** | BCL2-like 11 (apoptosis facilitator) | AY305715.1 |
| 4.774694 | **2.2554083** | up | **MMP13** | matrix metallopeptidase 13 (collagenase 3) | BC074807.2 |
| 4.7744055 | **2.255321** | up | **ARRDC4** | arrestin domain containing 4 | AK290221.1 |
| 4.7563024 | **2.2498405** | up | **SLC2A1** | solute carrier family 2 (facilitated glucose transporter), member 1 | NM_006516.2 |
| 4.7421727 | **2.2455482** | up | **ZNF295** | zinc finger protein 295 | AK300291.1 |
| 4.726506 | **2.2407742** | up | **FNDC5** | fibronectin type III domain containing 5 | NM_153756.1 |
| 4.718238 | **2.238248** | up | **ZFY** | zinc finger protein, Y-linked | NM_003411.3 |
| 4.6970706 | **2.2317612** | up | **FGFR1OP2** | FGFR1 oncogene partner 2 | BC032143.2 |
| 4.670982 | **2.2237258** | up | **CHM** | choroideremia (Rab escort protein 1) | NM_001145414.1 |
| 4.669155 | **2.2231615** | up | **ZXDA /// ZXDB** | zinc finger, X-linked, duplicated A /// zinc finger, X-linked, duplicated B | NM_007156.3 |
| 4.6642737 | **2.2216525** | up | **ZNF292** | zinc finger protein 292 | DA569554 |
| 4.642807 | **2.2149973** | up | **TBC1D1** | TBC1 (tre-2/USP6, BUB2, cdc16) domain family, member 1 | BC126979.1 |
| 4.6394377 | **2.21395** | up | **FAM189A2** | family with sequence similarity 189, member A2 | NM_004816.3 |
| 4.6014204 | **2.2020793** | up | **RSPH10B /// RSPH10B2** | radial spoke head 10 homolog B (Chlamydomonas) /// radial spoke head 10 homolog B2 (Chlamydomonas) | NM_173565.3 |
| 4.588392 | **2.1979885** | up | **PTP4A1** | protein tyrosine phosphatase type IVA, member 1 | BC045571.1 |
| 4.571434 | **2.1926467** | up | **RAB6A /// RAB6C /// WTH3DI** | RAB6A, member RAS oncogene family /// RAB6C, member RAS oncogene family /// RAB6C-like | AK301534.1 |
| 4.5658236 | **2.190875** | up | **SGMS2** | sphingomyelin synthase 2 | BC041369.2 |
| 4.5373273 | **2.1818428** | up | **CDKN2B** | cyclin-dependent kinase inhibitor 2B (p15, inhibits CDK4) | NM_078487.2 |
| 4.5352373 | **2.181178** | up | **SIPA1L2** | signal-induced proliferation-associated 1 like 2 | AI479863 |
| 4.519495 | **2.1761615** | up | **RNF38** | ring finger protein 38 | AK093480.1 |
| 4.508363 | **2.1726036** | up | **RGS3** | regulator of G-protein signaling 3 | AA609212 |
| 4.5062637 | **2.1719317** | up | **ZFX** | zinc finger protein, X-linked | M30608.1 |
| 4.47735 | **2.162645** | up | **CCDC11** | coiled-coil domain containing 11 | NM_145020.3 |
| 4.4671264 | **2.159347** | up | **RAB27B** | RAB27B, member RAS oncogene family | U57093.2 |
| 4.4564495 | **2.1558948** | up | **MEX3B** | mex-3 homolog B (C. elegans) | NM_032246.3 |
| 4.455477 | **2.15558** | up | **RIOK3** | RIO kinase 3 (yeast) | AK303938.1 |
| 4.416434 | **2.1428819** | up | **ZFX /// ZFY** | zinc finger protein, X-linked /// zinc finger protein, Y-linked | M30608.1 |
| 4.409302 | **2.1405504** | up | **DIRAS2** | DIRAS family, GTP-binding RAS-like 2 | BC008065.2 |
| 4.3955708 | **2.1360505** | up | **ICK** | intestinal cell (MAK-like) kinase | AF152469.1 |
| 4.351642 | **2.1215599** | up | **ARL14** | ADP-ribosylation factor-like 14 | NM_025047.2 |
| 4.344291 | **2.1191208** | up | **VPS37D** | vacuolar protein sorting 37 homolog D (S. cerevisiae) | NM_001077621.1 |
| 4.33884 | **2.1173093** | up | **RRM2B** | ribonucleotide reductase M2 B (TP53 inducible) | AB166669.1 |
| 4.331714 | **2.114938** | up | **MEF2A** | myocyte enhancer factor 2A | NM_005587.2 |
| 4.3297195 | **2.1142735** | up | **RNF145** | ring finger protein 145 | CK821117 |
| 4.323839 | **2.1123128** | up | **IDS** | iduronate 2-sulfatase | AA701678 |
| 4.295328 | **2.1027684** | up | **LAMP2** | lysosomal-associated membrane protein 2 | AK293931.1 |
| 4.289694 | **2.1008747** | up | **PCF11** | PCF11, cleavage and polyadenylation factor subunit, homolog (S. cerevisiae) | NM_015885.3 |
| 4.2840977 | **2.0989914** | up | **GNA13** | guanine nucleotide binding protein (G protein), alpha 13 | L22075.1 |
| 4.2819433 | **2.0982656** | up | **BRD2** | bromodomain containing 2 | BQ018124 |
| 4.28124 | **2.0980287** | up | **CHMP4C** | charged multivesicular body protein 4C | AY329086.1 |
| 4.2718534 | **2.0948622** | up | **UBL3** | ubiquitin-like 3 | CR533458.1 |
| 4.262033 | **2.0915418** | up | **SAP30** | Sin3A-associated protein, 30kDa | NM_003864.3 |
| 4.2508388 | **2.0877476** | up | **NUFIP2** | nuclear fragile X mental retardation protein interacting protein 2 | AK293075.1 |
| 4.2283444 | **2.080093** | up | **SGK1** | serum/glucocorticoid regulated kinase 1 | NM_001143676.1 |
| 4.2135773 | **2.0750456** | up | **MEX3C** | mex-3 homolog C (C. elegans) | AY950679.1 |
| 4.2090683 | **2.0735009** | up | **CSRNP1** | cysteine-serine-rich nuclear protein 1 | BM129310 |
| 4.2085466 | **2.073322** | up | **ZC2HC1A** | zinc finger, C2HC-type containing 1A | CD366135 |
| 4.1983566 | **2.0698247** | up | **SLC35G1** | solute carrier family 35, member G1 | DB314877 |
| 4.1978383 | **2.0696466** | up | **CEP63** | centrosomal protein 63kDa | AK056465.1 |
| 4.194388 | **2.0684602** | up | **FAM178A** | family with sequence similarity 178, member A | BC073832.1 |
| 4.1871924 | **2.0659833** | up | **CWF19L2** | CWF19-like 2, cell cycle control (S. pombe) | CR744485 |
| 4.1716943 | **2.0606334** | up | **STX11** | syntaxin 11 | BC033519.1 |
| 4.169483 | **2.0598686** | up | **RGS3** | regulator of G-protein signaling 3 | AK289666.1 |
| 4.1602354 | **2.0566652** | up | **SPECC1** | sperm antigen with calponin homology and coiled-coil domains 1 | NM_001033554.1 |
| 4.1557684 | **2.0551152** | up | **SREK1IP1** | SREK1-interacting protein 1 | DB260642 |
| 4.154305 | **2.0546072** | up | **STXBP5** | syntaxin binding protein 5 (tomosyn) | DB515190 |
| 4.149544 | **2.0529528** | up | **DDIT3** | DNA-damage-inducible transcript 3 | AV734189 |
| 4.148064 | **2.0524383** | up | **ESF1** | ESF1, nucleolar pre-rRNA processing protein, homolog (S. cerevisiae) | BU927851 |
| 4.1424646 | **2.0504894** | up | **ZNF644** | zinc finger protein 644 | BQ014639 |
| 4.1352916 | **2.0479891** | up | **NAIF1** | nuclear apoptosis inducing factor 1 | NM_197956.3 |
| 4.13493 | **2.047863** | up | **NDFIP1** | Nedd4 family interacting protein 1 | BG287742 |
| 4.11975 | **2.0425568** | up | **SERTAD1** | SERTA domain containing 1 | NM_013376.3 |
| 4.1127014 | **2.0400863** | up | **TPST1** | tyrosylprotein sulfotransferase 1 | CR611624.1 |
| 4.1119795 | **2.039833** | up | **RYBP** | RING1 and YY1 binding protein | BC014959.1 |
| 4.1048512 | **2.03733** | up | **SIK1** | salt-inducible kinase 1 | NM_173354.3 |
| 4.1014214 | **2.036124** | up | **TFAP2A** | transcription factor AP-2 alpha (activating enhancer binding protein 2 alpha) | BF343007 |
| 4.1010356 | **2.0359883** | up | **BARX2** | BARX homeobox 2 | NM_003658.4 |
| 4.0838103 | **2.0299158** | up | **IRX5** | iroquois homeobox 5 | U90304.1 |
| 4.080426 | **2.02872** | up | **MYCL1** | v-myc myelocytomatosis viral oncogene homolog 1, lung carcinoma derived (avian) | NM_001033081.1 |
| 4.077265 | **2.0276017** | up | **AZI2** | 5-azacytidine induced 2 | NM_001134433.1 |
| 4.0590067 | **2.0211267** | up | **FKBP1A-SDCBP2 /// SDCBP2** | FKBP1A-SDCBP2 readthrough (non-protein coding) /// syndecan binding protein (syntenin) 2 | NM_080489.3 |
| 4.048141 | **2.0172596** | up | **PCDH1** | protocadherin 1 | NM_002587.3 |
| 4.041768 | **2.0149865** | up | **RAET1L** | retinoic acid early transcript 1L | NM_130900.2 |
| 4.0390162 | **2.014004** | up | **ZSWIM3** | zinc finger, SWIM-type containing 3 | NM_080752.2 |
| 4.0172997 | **2.006226** | up | **CPEB4** | cytoplasmic polyadenylation element binding protein 4 | BC117150.1 |
| 4.004509 | **2.0016253** | up | **ZXDB** | zinc finger, X-linked, duplicated B | AK289766.1 |
