# Supplementary material for: Genome Wide Expression Profiling of Cancer Cell Lines Cultured in Microgravity Reveals Significant Dysregulation of Cell Cycle and MicroRNA Gene Networks
Source: PLoS One. 2015 Aug 21;10(8):e0135958. doi: 10.1371/journal.pone.0135958 (PMC4546578; doi:10.1371/journal.pone.0135958)
Supplement: S4 Table — (DOCX) [file pone.0135958.s005.docx]

| **> 2 log fold Up and down regulated genes in microarray of MOLT-4 cells under microgravity** | | | | | |
| --- | --- | --- | --- | --- | --- |
| **Fold Change**  **MOLT-4**  **RCCS Vs Static** | **Log Fold Change**  **MOLT-4**  **RCCS Vs Static** | **Reg.** | **Gene Symbol** | **Gene Title** | **Representative Public ID** |
| 61.06472 | **5.932267** | up | **EGR1** | early growth response 1 | NM_001964.2 |
| 15.18369 | **3.924451** | up | **EGR2** | early growth response 2 | NM_001136178.1 |
| 10.91651 | **3.448439** | up | **JUN** | jun proto-oncogene | AW008290 |
| 10.77003 | **3.42895** | up | **FOS** | FBJ murine osteosarcoma viral oncogene homolog | NM_005252.2 |
| 8.558331 | **3.097329** | up | **CD69** | CD69 molecule | NM_001781.2 |
| 7.586817 | **2.923495** | up | **SGK1** | serum/glucocorticoid regulated kinase 1 | NM_001143676.1 |
| 7.250296 | **2.85804** | up | **NR4A1** | nuclear receptor subfamily 4, group A, member 1 | NM_002135.3 |
| 6.337676 | **2.663954** | up | **NR4A3** | nuclear receptor subfamily 4, group A, member 3 | NM_173198.1 |
| 5.825823 | **2.542462** | up | **ARRDC3** | arrestin domain containing 3 | NM_020801.2 |
| 5.611444 | **2.488372** | up | **ETV5** | ets variant 5 | NM_004454.2 |
| 5.139436 | **2.36161** | up | **RNF125** | ring finger protein 125, E3 ubiquitin protein ligase | BC012021.1 |
| 4.644002 | **2.215369** | up | **SCN3B** | sodium channel, voltage-gated, type III, beta subunit | NM_018400.3 |
| 4.590877 | **2.19877** | up | **SCARNA14** | small Cajal body-specific RNA 14 | NR_004388.1 |
| 4.409986 | **2.140774** | up | **EGR4** | early growth response 4 | NM_001965.2 |
| **Fold Change**  **MOLT-4**  **RCCS Vs Static** | **Log Fold Change**  **MOLT-4**  **RCCS Vs Static** | **Reg.** | **Gene Symbol** | **Gene Title** | **Representative Public ID** |
| -5.55138 | **-2.47285** | down | **RBM8A** | RNA binding motif protein 8A | DB326990 |
| -5.28555 | **-2.40205** | down | **MIR17HG /// MIR18A /// MIR19A /// MIR19B1 /// MIR20A /// MIR92A1** | miR-17-92 cluster host gene (non-protein coding) /// microRNA 18a /// microRNA 19a /// microRNA 19b-1 /// microRNA 20a /// microRNA 92a-1 | AB176707.1 |
| -4.96984 | **-2.3132** | down | **PTPRF** | protein tyrosine phosphatase, receptor type, F | AB177856.1 |
| -4.54736 | **-2.18503** | down | **D4S234E** | DNA segment on chromosome 4 (unique) 234 expressed sequence | NM_001040101.1 |
| -4.51572 | **-2.17496** | down | **DNTT** | deoxynucleotidyltransferase, terminal | NM_004088.3 |
| -4.47263 | **-2.16112** | down | **MIR1304 /// SNORA1 /// SNORA18 /// SNORA32 /// SNORA40 /// SNORA8 /// SNORD5 /// TAF1D** | microRNA 1304 /// small nucleolar RNA, H/ACA box 1 /// small nucleolar RNA, H/ACA box 18 /// small nucleolar RNA, H/ACA box 32 /// small nucleolar RNA, H/ACA box 40 /// small nucleolar RNA, H/ACA box 8 /// small nucleolar RNA, C/D box 5 /// TATA box binding protein (TBP)-associated factor, RNA polymerase I, D, 41kDa | NR_002959.1 |
| -4.45893 | **-2.1567** | down | **NKAIN4** | Na+/K+ transporting ATPase interacting 4 | CN369492 |
| -4.41493 | **-2.14239** | down | **AGRN** | agrin | NM_198576.2 |
| -4.33353 | **-2.11554** | down | **RAG1** | recombination activating gene 1 | NM_000448.2 |
| -4.14512 | **-2.05141** | down | **B4GALNT4** | beta-1,4-N-acetyl-galactosaminyl transferase 4 | NM_178537.4 |
| -4.05391 | **-2.01931** | down | **HES4** | hairy and enhancer of split 4 (Drosophila) | NM_021170.3 |
