# Supplementary material for: Genome Wide Expression Profiling of Cancer Cell Lines Cultured in Microgravity Reveals Significant Dysregulation of Cell Cycle and MicroRNA Gene Networks
Source: PLoS One. 2015 Aug 21;10(8):e0135958. doi: 10.1371/journal.pone.0135958 (PMC4546578; doi:10.1371/journal.pone.0135958)
Supplement: S5 Table — (DOCX) [file pone.0135958.s006.docx]

**Microarray analysis reveals commonly deregulated genes under Microgravity**

| **UPREGULATED GENES** | | | | | | |  |
| --- | --- | --- | --- | --- | --- | --- | --- |
| **GENE SYMBOL** | **GENE NAME** | | | **Log FC DLD1** | | **Log FC MOLT 4** |  |
| **ACAP2** | ArfGAP with coiled-coil, ankyrin repeat and PH domains 2 | | | 1.3913777 | | 1.076808 |  |
| **ACBD5** | acyl-CoA binding domain containing 5 | | | 1.2621408 | | 1.1323233 |  |
| **AFTPH** | aftiphilin | | | 1.0259514 | | 1.0670457 |  |
| **ANKRD12** | ankyrin repeat domain 12 | | | 1.95575 | | 1.340133 |  |
| **ARRDC3** | arrestin domain containing 3 | | | 3.6286426 | | 2.5424619 |  |
| **ATF3** | activating transcription factor 3 | | | 2.6872826 | | 1.0043197 |  |
| **C18orf25** | chromosome 18 open reading frame 25 | | | 1.515207 | | 1.3546908 |  |
| **C9orf85** | chromosome 9 open reading frame 85 | | | 1.639885 | | 1.0997329 |  |
| **CCPG1** | cell cycle progression 1 | | | 1.5802538 | | 1.8111625 |  |
| **CDKN2AIP** | CDKN2A interacting protein | | | 1.6052608 | | 1.0670295 |  |
| **CDKN2D** | cyclin-dependent kinase inhibitor 2D (p19, inhibits CDK4) | | | 1.3287859 | | 1.0733795 |  |
| **CHIC2** | cysteine-rich hydrophobic domain 2 | | | 1.1411004 | | 1.0720868 |  |
| **CPEB4** | cytoplasmic polyadenylation element binding protein 4 | | | 2.006226 | | 1.2051146 |  |
| **CREBBP** | CREB binding protein | | | 1.6003945 | | 1.1786065 |  |
| **CREBRF** | CREB3 regulatory factor | | | 1.7348738 | | 1.2481394 |  |
| **CXCL3** | chemokine (C-X-C motif) ligand 3 | | | 1.6632779 | | 1.1129959 |  |
| **DDIT3** | DNA-damage-inducible transcript 3 | | | 2.0701203 | | 1.280817 |  |
| **DUSP8 /// GLUD1P3** | dual specificity phosphatase 8 /// glutamate dehydrogenase 1 pseudogene 3 | | | 1.0926304 | | 1.434655 |  |
| **EGR2** | early growth response 2 | | | 1.3966465 | | 3.9244506 |  |
| **ETS1** | v-ets erythroblastosis virus E26 oncogene homolog 1 (avian) | | | 1.3279192 | | 1.0439773 |  |
| **ETV5** | ets variant 5 | | | 1.2021747 | | 2.488372 |  |
| **FAM178A** | family with sequence similarity 178, member A | | | 2.0684602 | | 1.0132704 |  |
| **FAM190B** | family with sequence similarity 190, member B | | | 1.392551 | | 1.0767126 |  |
| **FAM214A** | family with sequence similarity 214, member A | | | 1.4883766 | | 1.0134716 |  |
| **FGF7 /// KGFLP1 /// KGFLP2** | fibroblast growth factor 7 /// fibroblast growth factor 7 pseudogene /// keratinocyte growth factor-like protein 2 | | | 2.2669797 | | 1.1106136 |  |
| **GORAB** | golgin, RAB6-interacting | | | 1.1204505 | | 1.0041857 |  |
| **HDAC9** | histone deacetylase 9 | | | 1.0828898 | | 1.4470437 |  |
| **HINT3** | histidine triad nucleotide binding protein 3 | | | 1.7376733 | | 1.7083709 |  |
| **HIST1H2BD** | histone cluster 1, H2bd | | | 3.5836997 | | 1.1623578 |  |
| **HIST2H2AA3 /// HIST2H2AA4** | histone cluster 2, H2aa3 /// histone cluster 2, H2aa4 | | | 1.5469661 | | 1.049464 |  |
| **HIVEP2** | human immunodeficiency virus type I enhancer binding protein 2 | | | 1.2518463 | | 1.2009206 |  |
| **INTS6** | integrator complex subunit 6 | | | 1.8347211 | | 1.0919981 |  |
| **IRS2** | insulin receptor substrate 2 | | | 2.5675535 | | 1.0355048 |  |
| **JUN** | jun proto-oncogene | | | 2.8459454 | | 3.4484391 |  |
| **KLHDC10** | kelch domain containing 10 | | | 1.769959 | | 1.9192705 |  |
| **KRCC1** | lysine-rich coiled-coil 1 | | | 1.1697567 | | 1.8856514 |  |
| **MIR1304 /// SNORA1 /// SNORA18 /// SNORA32 /// SNORA40 /// SNORA8 /// SNORD5 /// TAF1D** | microRNA 1304 /// small nucleolar RNA, H/ACA box 1 /// small nucleolar RNA, H/ACA box 18 /// small nucleolar RNA, H/ACA box 25 /// small nucleolar RNA, H/ACA box 32 /// small nucleolar RNA, H/ACA box 40 /// small nucleolar RNA, H/ACA box 8 /// small nucleolar RNA, C/D box 5 /// TATA box binding protein (TBP)-associated factor, RNA polymerase I, D, 41kDa | | | 1.694859 | | 1.0175457 |  |
| **NCOA7** | nuclear receptor coactivator 7 | | | 1.3721471 | | 1.0397696 |  |
| **NDFIP2** | Nedd4 family interacting protein 2 | | | 1.0812235 | | 1.1697233 |  |
| **NR4A3** | nuclear receptor subfamily 4, group A, member 3 | | | 3.2986097 | | 2.6639538 |  |
| **PIBF1** | progesterone immunomodulatory binding factor 1 | | | 1.6272297 | | 1.2976668 |  |
| **PLEKHF2** | pleckstrin homology domain containing, family F (with FYVE domain) member 2 | | | 2.5150435 | | 1.105514 |  |
| **PPP3R1** | protein phosphatase 3, regulatory subunit B, alpha | | | 2.3322349 | | 1.1869926 |  |
| **PRDM1** | PR domain containing 1, with ZNF domain | | | 1.402319 | | 1.2224886 |  |
| **PTEN** | phosphatase and tensin homolog | | | 1.2177935 | | 1.0146773 |  |
| **RAB30** | RAB30, member RAS oncogene family | | | 1.8227141 | | 1.6890364 |  |
| **RAET1G /// RAET1L /// ULBP2** | retinoic acid early transcript 1G /// retinoic acid early transcript 1L /// UL16 binding protein 2 | | | 1.496223 | | 1.0062566 |  |
| **RC3H1** | ring finger and CCCH-type domains 1 | | | 1.6937757 | | 1.1645081 |  |
| **RGS16** | regulator of G-protein signaling 16 | | | 3.0088909 | | 1.2928457 |  |
| **RNF32** | ring finger protein 32 | | | 1.6749833 | | 1.0492477 |  |
| **RNPC3** | RNA-binding region (RNP1, RRM) containing 3 | | | 1.4769745 | | 1.2600317 |  |
| **SAMD8** | sterile alpha motif domain containing 8 | | | 1.5586944 | | 1.2673981 |  |
| **SARNP** | SAP domain containing ribonucleoprotein | | | 1.3978903 | | 1.172842 |  |
| **SEC24A** | SEC24 family, member A (S. cerevisiae) | | | 1.3752923 | | 1.6501074 |  |
| **SGK1** | serum/glucocorticoid regulated kinase 1 | | | 2.080093 | | 2.9234948 |  |
| **SH3GLB1** | SH3-domain GRB2-like endophilin B1 | | | 1.2074189 | | 1.0221386 |  |
| **SKIL** | SKI-like oncogene | | | 1.7823422 | | 1.4903564 |  |
| **SMAD7** | SMAD family member 7 | | | 1.0359683 | | 1.338062 |  |
| **SMAP2** | small ArfGAP2 | | | 1.1966066 | | 1.1760144 |  |
| **SNHG12 /// SNORA16A /// SNORA44 /// SNORA61** | small nucleolar RNA host gene 12 (non-protein coding) /// small nucleolar RNA, H/ACA box 16A /// small nucleolar RNA, H/ACA box 44 /// small nucleolar RNA, H/ACA box 61 | | | 1.2712197 | | -1.5065367 |  |
| **SNRNP48** | small nuclear ribonucleoprotein 48kDa (U11/U12) | | | 1.1283903 | | 1.1029987 |  |
| **STK16** | serine/threonine kinase 16 | | | 1.2336359 | | 1.0671515 |  |
| **STRN** | striatin, calmodulin binding protein | | | 1.7833431 | | 1.0033815 |  |
| **TICAM2 /// TMED7-TICAM2** | toll-like receptor adaptor molecule 2 /// TMED7-TICAM2 readthrough | | | 1.6961193 | | 1.4189873 |  |
| **TNFAIP3** | tumor necrosis factor, alpha-induced protein 3 | | | 1.2988296 | | 1.3440051 |  |
| **TNKS** | tankyrase, TRF1-interacting ankyrin-related ADP-ribose polymerase | | | 1.1008911 | | 1.0766392 |  |
| **TRAPPC6B** | trafficking protein particle complex 6B | | | 1.5925066 | | 1.5012903 |  |
| **TRIB1** | tribbles homolog 1 (Drosophila) | | | 1.0343199 | | 1.0414665 |  |
| **UBXN7** | UBX domain protein 7 | | | 1.2349052 | | 1.2993069 |  |
| **ULBP2** | UL16 binding protein 2 | | | 1.527741 | | 1.1375837 |  |
| **XIAP** | X-linked inhibitor of apoptosis | | | 1.3545609 | | 1.2860351 |  |
| **ZFAND2A** | zinc finger, AN1-type domain 2A | | | 1.9506464 | | 1.2355728 |  |
| **ZFY** | zinc finger protein, Y-linked | | | 2.238248 | | 1.2044845 |  |
| **ZMYM5** | zinc finger, MYM-type 5 | | | 1.7800057 | | 1.4686751 |  |
| **ZNF425** | zinc finger protein 425 | | | 2.476612 | | 1.0105476 |  |
| **ZNF571** | zinc finger protein 571 | | | 1.4478207 | | 1.0791094 |  |
| **ZNF571** | zinc finger protein 571 | | | 1.4057643 | | 1.0791094 |  |
| **DOWNREGULATED GENES** | | | | | | | |
| **GENE**  **SYMBOL** | | **GENE NAME** | **Log FC**  **DLD1** | | **Log FC MOLT 4** | | |
| **ACBD7** | | acyl-CoA binding domain containing 7 | -1.7749069 | | -1.012526 | | |
| **ADAT2** | | adenosine deaminase, tRNA-specific 2 | -1.2610927 | | -1.0094724 | | |
| **AK2** | | adenylate kinase 2 | -1.3070328 | | -1.1065421 | | |
| **AKAP12** | | A kinase (PRKA) anchor protein 12 | -1.4494083 | | -1.1301394 | | |
| **ANP32A** | | acidic (leucine-rich) nuclear phosphoprotein 32 family, member A | -1.7761931 | | -1.5069108 | | |
| **ASIC1** | | acid-sensing (proton-gated) ion channel 1 | -1.1945584 | | -1.4278164 | | |
| **BAHCC1** | | BAH domain and coiled-coil containing 1 | -1.2710049 | | -1.413588 | | |
| **BRI3BP** | | BRI3 binding protein | -1.0864185 | | -1.495153 | | |
| **BTNL9** | | butyrophilin-like 9 | -1.6387166 | | -1.1642449 | | |
| **C4orf46** | | chromosome 4 open reading frame 46 | -2.2585483 | | -1.001637 | | |
| **CBX5** | | chromobox homolog 5 | -1.5238662 | | -1.0014915 | | |
| **CD24** | | CD24 molecule | -2.1321063 | | -1.3863628 | | |
| **CDCA7L** | | cell division cycle associated 7-like | -1.6832285 | | -1.0647273 | | |
| **CDKN1C** | | cyclin-dependent kinase inhibitor 1C (p57, Kip2) | -1.2500896 | | -1.0963018 | | |
| **CEP128** | | centrosomal protein 128kDa | -1.7307765 | | -1.1035609 | | |
| **CORO2A** | | coronin, actin binding protein, 2A | -1.08589 | | -1.048481 | | |
| **CPT1A** | | carnitine palmitoyltransferase 1A (liver) | -1.1249452 | | -1.2743046 | | |
| **CR2** | | complement component (3d/Epstein Barr virus) receptor 2 | -1.0533493 | | -1.2196734 | | |
| **CSTF3** | | cleavage stimulation factor, 3' pre-RNA, subunit 3, 77kDa | -1.8889158 | | -1.1470387 | | |
| **CTDSPL** | | CTD (carboxy-terminal domain, RNA polymerase II, polypeptide A) small phosphatase-like | -1.4872465 | | -1.3061688 | | |
| **CTSC** | | cathepsin C | -1.6970179 | | -1.3670623 | | |
| **CYB5B** | | cytochrome b5 type B (outer mitochondrial membrane) | -1.1004338 | | -1.2150698 | | |
| **DDX11 /// LOC642846** | | DEAD/H (Asp-Glu-Ala-Asp/His) box helicase 11 /// DEAD/H (Asp-Glu-Ala-Asp/His) box polypeptide 11-like | -1.3138216 | | -1.0397248 | | |
| **DHFR** | | dihydrofolate reductase | -1.8050942 | | -1.0276904 | | |
| **DNHD1** | | dynein heavy chain domain 1 | -1.464865 | | -1.0485542 | | |
| **DUT** | | deoxyuridine triphosphatase | -1.0620422 | | -1.175415 | | |
| **EEF1A1 /// LOC100653236** | | eukaryotic translation elongation factor 1 alpha 1 /// uncharacterized LOC100653236 | -1.0099685 | | -1.0678835 | | |
| **EIF4A1** | | eukaryotic translation initiation factor 4A1 | -1.0106502 | | -1.0274181 | | |
| **ELAVL1** | | ELAV (embryonic lethal, abnormal vision, Drosophila)-like 1 (Hu antigen R) | -1.0304809 | | -1.0112107 | | |
| **ENAH** | | enabled homolog (Drosophila) | -1.1342392 | | -1.4717889 | | |
| **ENSA** | | endosulfine alpha | -1.0821726 | | -1.1114485 | | |
| **FANCL** | | Fanconi anemia, complementation group L | -1.2304835 | | -1.0152798 | | |
| **FAR1** | | fatty acyl CoA reductase 1 | -1.0487332 | | -1.0339952 | | |
| **FGFR3** | | fibroblast growth factor receptor 3 | -1.6760249 | | -1.3410914 | | |
| **GCSH** | | glycine cleavage system protein H (aminomethyl carrier) | -1.1946657 | | -1.3082767 | | |
| **GCSH /// LOC729080** | | glycine cleavage system protein H (aminomethyl carrier) /// glycine cleavage system protein H (aminomethyl carrier) pseudogene | -1.364049 | | -1.03936 | | |
| **GGH** | | gamma-glutamyl hydrolase (conjugase, folylpolygammaglutamyl hydrolase) | -1.2075133 | | -1.0368428 | | |
| **GINS2** | | GINS complex subunit 2 (Psf2 homolog) | -3.0693526 | | -1.0141101 | | |
| **GJA9-MYCBP /// MYCBP** | | GJA9-MYCBP readthrough /// c-myc binding protein | -1.2446318 | | -1.0228753 | | |
| **GSPT1** | | G1 to S phase transition 1 | -1.3995273 | | -1.0519781 | | |
| **GSTA4** | | glutathione S-transferase alpha 4 | -1.1619577 | | -1.0276983 | | |
| **HES4** | | hairy and enhancer of split 4 (Drosophila) | -1.1826415 | | -2.0193136 | | |
| **HMGB1** | | high mobility group box 1 | -1.002748 | | -1.2596858 | | |
| **HMGB3** | | high mobility group box 3 | -1.2634602 | | -1.1885982 | | |
| **HNRNPR** | | heterogeneous nuclear ribonucleoprotein R | -1.0010815 | | -1.3932297 | | |
| **HNRNPU** | | heterogeneous nuclear ribonucleoprotein U (scaffold attachment factor A) | -1.2706835 | | -1.1723645 | | |
| **HNRPDL** | | heterogeneous nuclear ribonucleoprotein D-like | -1.7951047 | | -1.7009487 | | |
| **HSPA4** | | heat shock 70kDa protein 4 | -1.5835454 | | -1.2644181 | | |
| **HSPA8 /// SNORD14C /// SNORD14D** | | heat shock 70kDa protein 8 /// small nucleolar RNA, C/D box 14C /// small nucleolar RNA, C/D box 14D | -1.2667797 | | -1.0284889 | | |
| **IFI30** | | interferon, gamma-inducible protein 30 | -1.5613956 | | -1.5471194 | | |
| **IFRD2** | | interferon-related developmental regulator 2 | -1.1622665 | | -1.0979714 | | |
| **IMPA2** | | inositol(myo)-1(or 4)-monophosphatase 2 | -1.5216382 | | -1.479593 | | |
| **JPH1** | | junctophilin 1 | -1.1266716 | | -1.6309524 | | |
| **KIAA0101** | | KIAA0101 | -1.0846858 | | -1.3091683 | | |
| **KIAA0664** | | KIAA0664 | -1.1195109 | | -1.0255272 | | |
| **KIF26A** | | kinesin family member 26A | -1.9151876 | | -1.4613476 | | |
| **KLHL23 /// PHOSPHO2-KLHL23** | | kelch-like 23 (Drosophila) /// PHOSPHO2-KLHL23 readthrough | -1.4257302 | | -1.1481166 | | |
| **LOC100288602 /// PPIA** | | peptidyl-prolyl cis-trans isomerase A-like /// peptidylprolyl isomerase A (cyclophilin A) | -1.2413359 | | -1.147387 | | |
| **LOC100507645 /// MALAT1** | | uncharacterized LOC100507645 /// metastasis associated lung adenocarcinoma transcript 1 (non-protein coding) | -1.012897 | | -1.5750542 | | |
| **LOC100653301 /// NRBP2** | | nuclear receptor-binding protein 2-like /// nuclear receptor binding protein 2 | -1.5101182 | | -1.0871661 | | |
| **MAT2A** | | methionine adenosyltransferase II, alpha | -1.3023586 | | -1.4851573 | | |
| **MCM2** | | minichromosome maintenance complex component 2 | -1.6556995 | | -1.0456934 | | |
| **MCM4** | | minichromosome maintenance complex component 4 | -2.1306236 | | -1.1940293 | | |
| **MRPS25** | | mitochondrial ribosomal protein S25 | -1.1645536 | | -1.3681169 | | |
| **MTPAP** | | mitochondrial poly(A) polymerase | -2.2048273 | | -1.046771 | | |
| **NEURL1B** | | neuralized homolog 1B (Drosophila) | -1.2790649 | | -1.0592682 | | |
| **NFIA** | | nuclear factor I/A | -1.2247176 | | -1.1426408 | | |
| **NLN** | | neurolysin (metallopeptidase M3 family) | -1.5272717 | | -1.3974905 | | |
| **NREP** | | neuronal regeneration related protein homolog (rat) | -1.4269035 | | -1.0265727 | | |
| **NSUN5P1 /// NSUN5P2** | | NOP2/Sun domain family, member 5 pseudogene 1 /// NOP2/Sun domain family, member 5 pseudogene 2 | -1.3127828 | | -1.1815896 | | |
| **NSUN5P2** | | NOP2/Sun domain family, member 5 pseudogene 2 | -1.6129994 | | -1.009675 | | |
| **NT5DC2** | | 5'-nucleotidase domain containing 2 | -1.1637392 | | -1.5625048 | | |
| **PARP1** | | poly (ADP-ribose) polymerase 1 | -1.6084354 | | -1.0683279 | | |
| **PHKA1** | | phosphorylase kinase, alpha 1 (muscle) | -1.4648097 | | -1.3255553 | | |
| **PLXNA1** | | plexin A1 | -1.1962962 | | -1.3295693 | | |
| **PNPT1** | | polyribonucleotide nucleotidyltransferase 1 | -1.3692455 | | -1.0695791 | | |
| **POLR3H** | | polymerase (RNA) III (DNA directed) polypeptide H (22.9kD) | -1.0398519 | | -1.1780653 | | |
| **PPP2R5E** | | protein phosphatase 2, regulatory subunit B', epsilon isoform | -1.058049 | | -1.226798 | | |
| **PROS1** | | protein S (alpha) | -1.067625 | | -1.4008534 | | |
| **PRTFDC1** | | phosphoribosyl transferase domain containing 1 | -1.8294299 | | -1.0906208 | | |
| **PTP4A2** | | protein tyrosine phosphatase type IVA, member 2 | -1.1657519 | | -1.1398048 | | |
| **RBBP4** | | retinoblastoma binding protein 4 | -1.0829744 | | -1.1924767 | | |
| **RCN2** | | reticulocalbin 2, EF-hand calcium binding domain | -1.1394019 | | -1.0305963 | | |
| **SLC39A10** | | solute carrier family 39 (zinc transporter), member 10 | -2.5216508 | | -1.1428666 | | |
| **SLC6A6** | | solute carrier family 6 (neurotransmitter transporter, taurine), member 6 | -1.3915293 | | -1.0010033 | | |
| **SMARCA4** | | SWI/SNF related, matrix associated, actin dependent regulator of chromatin, subfamily a, member 4 | -1.2176757 | | -1.1001191 | | |
| **SMC1A** | | structural maintenance of chromosomes 1A | -1.8601036 | | -1.2168303 | | |
| **SNHG12 /// SNORA16A /// SNORA44 /// SNORA61** | | small nucleolar RNA host gene 11 (non-protein coding) /// small nucleolar RNA, H/ACA box 39 /// small nucleolar RNA, H/ACA box 60 | -1.5184448 | | -1.5065367 | | |
| **SNRPD1** | | small nuclear ribonucleoprotein D1 polypeptide 16kDa | -1.6701465 | | -1.0375495 | | |
| **SRSF1** | | serine/arginine-rich splicing factor 1 | -1.0994473 | | -1.1973615 | | |
| **SRSF11** | | serine/arginine-rich splicing factor 11 | -2.10018 | | -1.2000039 | | |
| **SRSF2** | | serine/arginine-rich splicing factor 2 | -1.3869429 | | -1.233274 | | |
| **SRSF3** | | serine/arginine-rich splicing factor 3 | -1.2103834 | | -1.0214 | | |
| **SRSF5** | | serine/arginine-rich splicing factor 5 | -1.1802144 | | -1.025496 | | |
| **SUGP2** | | SURP and G patch domain containing 2 | -1.1560097 | | -1.0919304 | | |
| **SYNCRIP** | | synaptotagmin binding, cytoplasmic RNA interacting protein | -1.1416254 | | -1.5985253 | | |
| **TTLL12** | | tubulin tyrosine ligase-like family, member 12 | -1.0103106 | | -1.0247827 | | |
| **TUBB** | | tubulin, beta class I | -1.1920624 | | -1.0390439 | | |
| **TXLNG2P** | | taxilin gamma 2, pseudogene | -1.5355744 | | -1.2157655 | | |
| **UHRF1** | | ubiquitin-like with PHD and ring finger domains 1 | -1.7097101 | | -1.1847544 | | |
| **USP46** | | ubiquitin specific peptidase 46 | -1.1504021 | | -1.0139122 | | |
| **WHSC1** | | Wolf-Hirschhorn syndrome candidate 1 | -1.1641781 | | -1.0915036 | | |
| **ZMYND19** | | zinc finger, MYND-type containing 19 | -1.0200996 | | -1.0501456 | | |
| **ZNRF1** | | zinc and ring finger 1, E3 ubiquitin protein ligase | -1.3250463 | | -1.0257716 | | |
